# Interleukin-35 administration counteracts established murine type 1 diabetes – possible involvement of regulatory T cells

**DOI:** 10.1038/srep12633

**Published:** 2015-07-30

**Authors:** Kailash Singh, Erik Kadesjö, Julia Lindroos, Marcus Hjort, Marcus Lundberg, Daniel Espes, Per-Ola Carlsson, Stellan Sandler, Lina Thorvaldson

**Affiliations:** 1 Department of Medical Cell Biology, Biomedical Centre, Uppsala University, Uppsala, Sweden; 2 Department of Medical Sciences, Uppsala University, Uppsala, Sweden

## Abstract

The anti-inflammatory cytokine IL-35 is produced by regulatory T (Treg) cells to suppress autoimmune and inflammatory responses. The role of IL-35 in type 1 diabetes (T1D) remains to be answered. To elucidate this, we investigated the kinetics of Treg cell response in the multiple low dose streptozotocin induced (MLDSTZ) T1D model and measured the levels of IL-35 in human T1D patients. We found that Treg cells were increased in MLDSTZ mice. However, the Treg cells showed a decreased production of anti-inflammatory (IL-10, IL-35, TGF-β) and increased pro-inflammatory (IFN-γ, IL-2, IL-17) cytokines, indicating a phenotypic shift of Treg cells under T1D condition. IL-35 administration effectively both prevented development of, and counteracted established MLDSTZ T1D, seemingly by induction of Eos expression and IL-35 production in Treg cells, thus reversing the phenotypic shift of the Treg cells. IL-35 administration reversed established hyperglycemia in NOD mouse model of T1D. Moreover, circulating IL-35 levels were decreased in human T1D patients compared to healthy controls. These findings suggest that insufficient IL-35 levels play a pivotal role in the development of T1D and that treatment with IL-35 should be investigated in treatment of T1D and other autoimmune diseases.

Type 1 diabetes (T1D) is etiologically considered to be an autoimmune disease^1^, where infiltration of innate and adaptive immune cells destroy the pancreatic β-cells, leading to development of T1D^1^,^2^,^3^. Emerging evidence suggests that human T1D, like other autoimmune diseases, e.g. rheumatoid arthritis and multiple sclerosis, is caused by a failure of immune tolerance as a result of a functional defect of the regulatory (Treg) cells^4^,^5^,^6^,^7^.

Treg cells are essential for controlling the immune system in order to prevent both autoimmune and inflammatory diseases. These cells are characterized by the expression of the transcription factor Foxp3, and in the absence of Foxp3 both mice and humans develop autoimmune diseases^8^,^9^,^10^,^11^,^12^,^13^. There are two subsets of Treg cells that maintain the central and peripheral tolerance; thymic derived (tTreg) and peripherally induced Treg (pTreg) cells^14^. To regulate the immunological tolerance, Treg cells use a variety of mechanisms^15^. Under inflammatory and autoimmune conditions, Treg cell should secrete anti-inflammatory cytokines such as interleukin-10 (IL-10), IL-35, and transforming growth factor-beta (TGF-β) in order to counteract the autoimmune immune attack^15^. However, recent reports suggest that Treg cells instead acquire a T effector cell phenotype and become “reprogrammed” into T helper (Th) like cells^16^,^17^. Phenotypically shifted Treg cells secrete pro-inflammatory cytokines such as interferon-gamma (IFN-γ) and IL-17a instead of anti-inflammatory cytokines, and could then paradoxically accelerate the autoimmune and inflammatory conditions^18^,^19^. Increased numbers of phenotypically shifted Treg cells, which have lost their suppressive function, have been reported in chronic infections, autoimmune diseases and upon allograft rejection^20^,^21^,^22^,^23^,^24^. Marwaha *et al*. have also reported that Treg cells acquire a Th17 like phenotype in human T1D^25^.

Pan *et al*. have shown that the Ikaros transcription family member Eos, together with Foxp3, is essential for maintaining the suppressive function of Treg cells^26^, but the role of Eos has not yet been studied in autoimmune and infectious diseases. Furthermore, it is not yet clear whether tTreg and/or pTreg cells switch their phenotype under autoimmune and inflammatory conditions. The kinetics of tTreg and pTreg cells in the early development of autoimmune and inflammatory diseases has not been studied. Likewise, the kinetics of the novel anti-inflammatory cytokine IL-35 in autoimmune diseases, e.g. T1D is still unclear. These unresolved questions urged us to examine and clarify the role of Treg cells during the early development of T1D, using the murine multiple low dose streptozotocin (MLDSTZ) induced T1D model^27^, the NOD mouse model, and analysis of human peripheral blood obtained from T1D patients and healthy control subjects.

## Results

### The numbers of Treg cells are increased in MLDSTZ induced T1D

**Figure 1:**
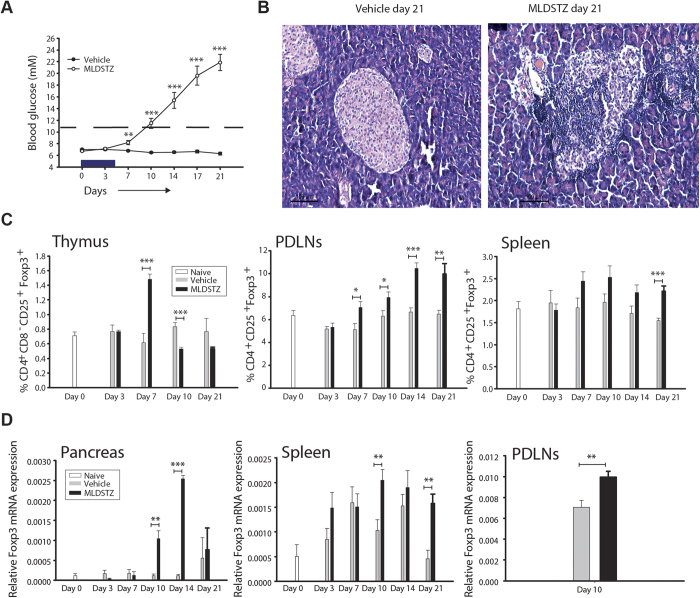
Treg cells are increased in MLDSTZ induced T1D. (**A**) Male CD-1 mice were injected i.p. with STZ (40 mg/kg/day) or 200 μl saline, for 5 consecutive days. The blood glucose levels (mM) were monitored in vehicle (closed circles) or MLDSTZ (open circles) treated mice on days 0, 3, 7, 10, 14, 17 and 21 after the first injection of STZ. (**B**) Representative light micrograph of pancreatic islets from vehicle (left panel) or MLDSTZ (right panel) treated mice on day 21 (hematoxylin and eosin staining; magnification ×100). (**C**) The proportions of CD4^+^CD25^+^Foxp3^+^ Treg cells in thymocytes, PDLNs cells and splenocytes were analyzed by flow cytometry in vehicle or MLDSTZ treated mice on days 0–21. (**D**) Real time RT-PCR analysis of the Foxp3 mRNA expression in pancreas, splenocytes and PDLN of vehicle or MLDSTZ treated mice was performed as described in the Methods section. Results are expressed as means ± SEM, from two experiments (n = 3 mice/group/experiment). Unpaired t-tests (a, c, e; spleen and PDLNs) and Wilcoxson Rank Sum tests (e; pancreas) were performed for comparisons between vehicle and MLDSTZ treated groups on corresponding days. *, ** and *** denote p < 0.05, p < 0.01, and p < 0.001, respectively.

MLDSTZ treated mice showed an increase in blood glucose levels from day 7, and became hyperglycemic from day 10, whilst saline treated (vehicle) mice remained normoglycemic (Fig. 1A). Pancreata of MLDSTZ showed insulitis from day 7, and the severity of insulitis gradually increased (Fig. 1B and Supplementary Table 1). The absolute numbers of leukocytes were also increased in pancreatic draining lymph nodes (PDLNs) and spleens of MLDSTZ treated mice (data not shown). The proportions of CD4^+^CD25^+^Foxp3^+^ Treg cells in MLDSTZ treated mice were increased on day 7 in the thymus, from day 7 in PDLNs, and on day 21 in spleen (Fig. 1C and Supplementary Fig. 1A). However, the proportion of Foxp3^+^ Treg cells was decreased on day 10 in the thymic glands of MLDSTZ mice (Fig. 1C).

The numbers of Foxp3^+^ cells were increased in the MLDSTZ treated group; from day 7 in pancreatic islets, and from day 10 in exocrine pancreas (Supplementary Table 1 and supplementary Fig. 2). Furthermore, the numbers of Foxp3^+^ cells were increased on day 21 in the red pulp of the spleen, from day 14 in white pulp, and in whole spleen (red + white pulp) from day 14 in MLDSTZ treated mice (Supplementary Fig. 1C). The relative expression levels of Foxp3 mRNA were increased on days 10 and 14 (pancreas), 10 and 21 (spleen), and on day 10 (PDLNs) in MLDSTZ treated mice (Fig. 1D).

Kiniwa *et al*. have shown that CD8^+^Foxp3^+^ T cells are immunosuppressive^28^, and we found that the proportion of CD8^+^Foxp3^+^ T cells was increased in thymic glands (on day 7) and PDLNs (on days 7 and 21) of MLDSTZ treated mice (Supplementary Fig. 1B and D). Taken together, our data demonstrate that both CD4^+^Foxp3^+^ and CD8^+^Foxp3^+^ Treg cells are increased in MLDSTZ induced T1D in mice.

### Both tTreg and pTreg cells are increased in MLDSTZ induced T1D

Next, we analyzed the proportions of tTreg cells and pTreg cells in thymic glands, PDLNs and spleen after MLDSTZ treatment. The expression of Helios (an Ikaros transcription factor family member) was used to distinguish between tTreg and pTreg cells^29^. The proportions of Helios^+^ tTreg cells were increased in thymus (on day 7), PDLNs (from day 7), and spleen (on day 21) of MLDSTZ treated mice (Supplementary Fig. 3A). Also, the proportions of Helios^−^ pTreg cells were increased in PDLNs and spleen of MLDSTZ treated mice from day 10 (Supplementary Fig. 3B).

It has been reported that Neuropilin-1 (Nrp1) also can be used as a marker for distinguishing tTreg from pTreg cells under certain conditions^30^,^31^. Therefore, we analyzed the proportions of Nrp1^+^ tTreg and Nrp1^−^ pTreg cells in vehicle and MLDSTZ treated mice. The proportions of Nrp1^+^ tTreg cells were increased on day 7 in thymus, from day 7 in PDLNs, and from day 10 in the spleen of MLDSTZ treated mice (Supplementary Fig. 3C). The proportions of Nrp1^−^ pTreg cells were increased in thymus (day 7) and PDLNs (from day 7) in MLDSTZ treated mice (Supplementary Fig. 3D). Recently, we have found that Nrp1 was not an optional marker for detection of tTreg cells in naïve mice^32^. We therefore also analyzed the proportions of Helios^+^Nrp1^+^ and Helios^+^Nrp1^−^ Treg cells, and found that the proportions of these cells were also increased in thymic glands, PDLNs and spleen of MLDSTZ mice (data not shown). Collectively, these results indicate that both tTreg and pTreg cells are increased in MLDSTZ induced T1D.

### Decreased production of IL-10, IL-35 and TGF-β by Treg cells in MLDSTZ induced T1D

We found that the increase of Treg cells failed to counteract the hyperglycemia in MLDSTZ mice (Fig. 1A), which is in line with observations from recent onset human T1D^25^. To further investigate this, the relative mRNA expression levels of anti-inflammatory Treg cytokines (IL-35, IL-10 and TGF-β)^15^ were determined on day 10 (when the mice were diabetic). IL-35 consists of two subunits; IL-12 alpha (IL-12p35 or IL-12a) and the Epstein-Barr-virus-induced gene (Ebi3)^33^, which were measured separately. The relative IL-12p35 and Ebi3 mRNA expressions were unchanged in PDLNs (on day 10) and spleen (on day 21) of MLDSTZ treated mice. Similarly, the relative mRNA expression level of Ebi3 was not altered in the pancreas of MLDSTZ treated mice on day 10 (Supplementary Fig. 4A–B). The IL-10 mRNA expression was decreased on day 10 in PDLNs of MLDSTZ treated mice, but not on day 21 in spleen (Supplementary Fig. 4C). TGF-β mRNA expression did not change in PDLNs and spleen after MLDSTZ (Supplementary Fig. 4D).

**Figure 2:**
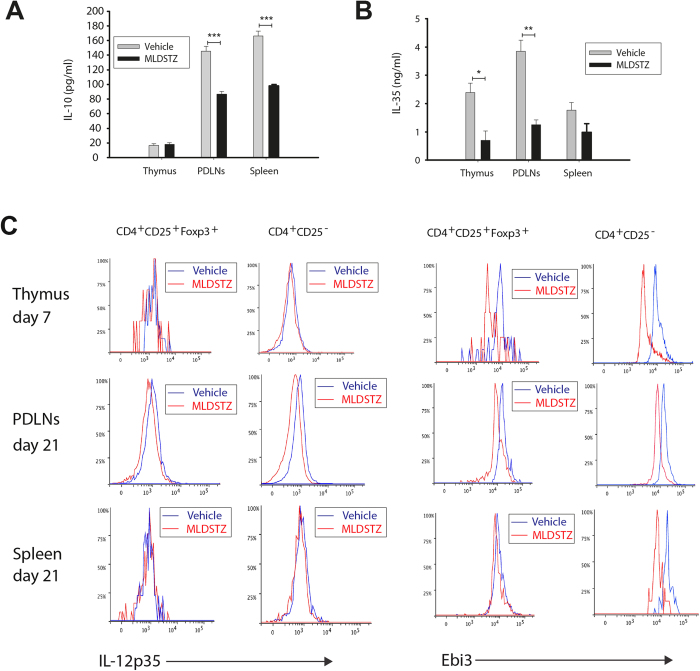
Impaired production of IL-10 and IL-35 by Treg cells in MLDSTZ induced T1D. (**A**) Concentrations of IL-10 and (**B**) IL-35 in supernatants of stimulated CD4^+^CD25^+^ Treg cells sorted from thymic glands, PDLNS and spleen of vehicle or MLDSTZ treated mice. (**C**) Representative histograms showing the expression of IL-12p35 (left panels) or Ebi3 (right panels) in CD4^+^CD25^+^Foxp3^+^ Treg or CD4^+^CD25^−^ Th cells in thymocytes, PDLNs cells and splenocytes of vehicle or MLDSTZ injected mice on indicated days. Results are expressed as means ± SEM, from two experiments (n = 3 mice/group/experiment). Unpaired t-tests were performed for comparisons between vehicle and MLDSTZ treated groups on corresponding days. *, ** and *** denote p < 0.05, p < 0.01, and p < 0.001, respectively.

In addition, the concentration of IL-10 was decreased in supernatants of stimulated CD4^+^CD25^+^ Treg cells from PDLNs and spleen, but not in supernatants of stimulated CD4^+^CD25^+^ Treg cells from thymic glands of MLDSTZ treated mice (Fig. 2A). Also, the IL-35 concentration was significantly decreased in the supernatant of stimulated CD4^+^CD25^+^ Treg cells from thymic glands and PDLNs of MLDSTZ mice compared to vehicle treated mice (Fig. 2B). However, we did not find any significant differences in the IL-35 concentration in supernatants from CD4^+^CD25^+^ Treg cells of spleen (Fig. 2B). The concentrations of TGF-β were 305 ± 3, 261 ± 14 and 316 ± 7 pg/ml in supernatants from CD4^+^CD25^+^ Treg cells of thymic glands, PDLNs and spleen of MLDSTZ mice, respectively. The concentrations of TGF-β were >500 pg/ml in all supernatants from CD4^+^CD25^+^ Treg cells of thymic glands, PDLNs and spleen of vehicle treated mice (data not shown). These results demonstrate that Treg cells produce lower amounts of anti-inflammatory cytokines under T1D-like conditions in MLDSTZ treated mice.

To further study if the production of IL-35 by Treg cells was failing after MLDSTZ treatment, the expression of Ebi3 and IL-12p35 in Foxp3^+^ Treg cells was investigated using flow cytometry, on days 7,10 and 21. The mean fluorescence intensity (MFI) of Ebi3 and IL-12p35 were decreased in Foxp3^+^ Treg cells of PDLNs of MLDSTZ mice (Fig. 2C and Supplementary Fig. 4E and F). The MFI of Ebi3 was also decreased in Foxp3^+^ Treg cells of thymic glands and spleen of MLDSTZ mice (Fig. 2C and Supplementary Fig. 4F).

Next, to further examine if the pancreatic Treg cells are producing/secreting enough IL-35, we counted the numbers of Ebi3-positive cells in pancreatic tissue from MLDSTZ treated mice on days 7 and 21. We did not find any Ebi3-positive cells in pancreas of MLDSTZ mice (data not shown) at the same time that we found an increase in the numbers of Foxp3^+^ cells. The fact that we could not detect any Ebi3^+^ cells suggest either that the Foxp3^+^ cells of MLDSTZ pancreases do not produce IL-35, or that the half-life of IL-35 in pancreas is very short, making detection difficult.

Collison *et al*. have shown that IL-35 can convert conventional T (Tconv) cells into IL-35 producing regulatory cells, designated iT_R_35 cells^34^. Therefore, the MFIs of Ebi3 and IL-12p35 in CD4^+^CD25^−^ T cells were analyzed. The MFI of Ebi3 in CD4^+^CD25^−^ T cells was decreased in thymic glands, PDLNs and spleen (Fig. 2C). The MFI of IL-12p35 in CD4^+^CD25^−^ T cell was unchanged in thymic glands and spleen, but lowered in PDLNs of MLDSTZ treated mice (Fig. 2C).

All together, our data indicate that the Treg cells have an impaired production of IL-10, IL-35 and TGF-β, which did not increase in response to MLDSTZ treatment irrespective of the fact that the numbers of Treg cells were increased.

### tTreg and pTreg cells undergo a phenotypic shift in MLDSTZ induced T1D

**Figure 3:**
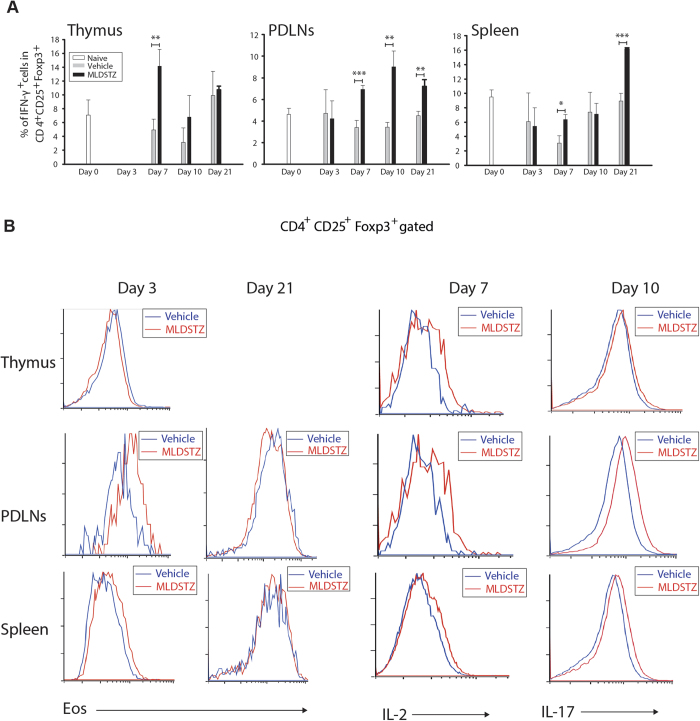
Treg cells acquire a T effector cell phenotype in MLDSTZ T1D. (**A**) The percentage of IFN-γ^+^ cells in Foxp3^+^ Treg cells of thymic glands, PDLNs and spleen of vehicle and MLDSTZ injected mice (gating strategies are shown in Supplementary Fig. 5A). (**B**) Representative histograms showing the expression of Eos, IL-2, and IL-17 in CD4^+^CD25^+^Foxp3^+^ Treg cells. Results are expressed as means ± SEM, from two experiments (n = 3 mice/group/experiment). Unpaired t-tests were performed for comparisons between vehicle and MLDSTZ treated mice. *, ** and *** denote p < 0.05, p < 0.01, and p < 0.001, respectively.

We subsequently investigated the impaired production of IL-35, IL-10 and TGF-β, and hypothesized that Treg cells in MLDSTZ induced T1D have changed their phenotype. Indeed, it has been reported that Treg cells can shift their phenotype under inflammatory conditions by starting to produce IFN-γ or IL-17^20^,^21^,^23^,^35^. Interestingly, we found that the percentage of IFN-γ expressing Foxp3^+^ Treg cells was increased on day 7 in thymus, from day 7 in PDLNs, and on days 7 and 21 in the spleen of MLDSTZ treated mice (Fig. 3A and Supplementary Fig. 5A). Thereafter, we examined whether it was the tTreg and/or the pTreg cell subset that had acquired a Th1 phenotype. The percentage of IFN-γ expressing Helios^+^ tTreg cells was increased in thymus (day 7), PDLNs (from day 7), and spleen (on days 7 and 21) of MLDSTZ treated mice (Supplementary Fig. 5B). In addition, the percentage of IFN-γ expressing Helios^−^ pTreg cells was also increased in PDLNs from day 10 and on day 21 in spleen of MLDSTZ treated mice (Supplementary Fig. 5C).

Rubtsov *et al*. have reported that the Foxp3 expression is stable in different disease models^36^. Since Eos together with Foxp3 maintains the suppressive phenotype of Treg cells by blocking IL-2 gene signaling in Treg cells^26^, we therefore analyzed the expression of Eos in Foxp3^+^ Treg cells, The Eos expression was decreased in Foxp3^+^ Treg cells in thymus (Fig. 3B, p < 0.05, not indicated in the figure), increased in PDLNs and spleen on day 3 (before the mice were showing any clear signs of leukocyte infiltration in pancreas) and impaired on days 10 and 21 in PDLNs and spleen in MLDSTZ treated mice (Fig. 3B). In addition, IL-2 expression was increased on day 7 in Foxp3^+^ Treg cells of thymus, PDLNs and spleen of MLDSTZ treated mice (Fig. 3B, p < 0.05, not indicated in the figures). Moreover, IL-17 expression was increased in Foxp3^+^ Treg cells (Fig. 3B, p < 0.05, not indicated in the figures). Recently, Foxp3^+^Eos^−^ Treg cells have been characterized as a bifunctional Treg cell that can change their phenotype under inflammatory conditions and this subset of Treg cells also express CD38^37^. Notably, both Foxp3^+^Eos^−^ and Foxp3^+^Eos^−^ CD38^+^ Treg cells were increased on day 21 in PDLNs and spleens of MLDSTZ treated mice (Supplementary Fig. 6A–B). Our results illustrate that both tTreg and pTreg cells change their phenotype during the early development of T1D, possibly because of impaired Eos expression in Foxp3^+^ Treg cells during the disease progression.

### Increased numbers of Treg cells fail to keep the numbers of CD4^+^CD25^−^ T helper (Th) and CD4^+^IL-17a^+^ (Th17) cells down in MLDSTZ induced T1D

**Figure 4:**
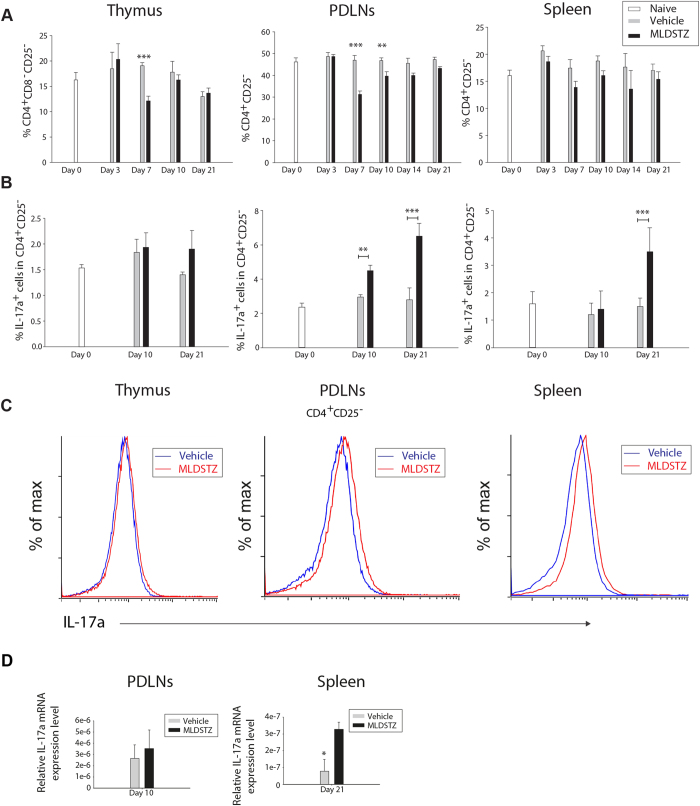
Increased numbers of tTreg and pTreg cells can not keep the numbers of Th17 cells down in MLDSTZ induced T1D. (**A**) CD4^+^CD25^−^ Th cells were analyzed by flow cytometry in thymic glands, PDLNs and spleen of vehicle and MLDSTZ injected mice. The proportions of CD4^+^CD25^−^ Th cells in total was expressed as means ± SEM. All significant differences in PDLN cells are not indicated in the figure, but they are, however, described in the Results section. (**B**) The percentage of IL-17a^+^ T cells in CD4^+^CD25^−^ Th cells were analyzed by flow cytometry in thymic glands, PDLNs and spleen of vehicle and MLDSTZ injected mice. (**C**) Representative histograms showing the expression of IL-17a in CD4^+^CD25^−^ Th cells. (**D**) Relative IL-17a mRNA expression in PDLN cells (on day 10) and splenocytes (on day 21) of vehicle or MLDSTZ injected mice were analyzed by real time RT-PCR. Results are expressed as means ± SEM, from two experiments (n = 3 mice/group/experiment). Unpaired t-tests were performed for comparisons between vehicle and MLDSTZ treated groups on corresponding days. ** and *** denote p < 0.01, and p < 0.001, respectively.

Our results indicate that Treg cells switch their phenotype in MLDSTZ induced T1D (Fig. 3) to non-suppressive IFN-γ producing Foxp3^+^ Treg cells. Therefore, we investigated the proportions of CD4^+^CD25^−^ Th and CD4^+^IL-17a^+^ (Th17) cells in thymus, PDLNs and spleen by flow cytometry, since functionally suppressive/active Treg cells keep the numbers of CD4^+^CD25^−^ Th cells and Th17 cells down both *in vitro* and *in vivo*^15^. In line with this hypothesis, Tang *et al*. have shown that phenotypically shifted Treg cells in the NOD mouse model are not functionally active/suppressive^20^. In addition, T1D has been associated with increased numbers of Th1 cells; however, Th17 cells also play a pivotal role in disease development^25^,^38^. The proportions of Th cells were decreased on day 7 in thymus and on days 7 and 10 in PDLNs of MLDSTZ treated mice (Fig. 4A). Moreover, the proportions of Th cells were increased on day 10 and onwards, compared to day 7 in PDLNs of MLDSTZ treated mice (Fig. 4B, not indicated, p < 0.001). In addition, the proportions of Th17 cells were increased from day 10 in PDLNs, and on day 21 in spleen of MLDSTZ mice (Fig. 4B,C). The relative IL-17a mRNA expression was increased in the spleen of MLDSTZ treated mice on day 21 (Fig. 4D). In conclusion, our results illustrate that the increase in tTreg and pTreg cells could not keep the numbers of CD4^+^CD25^−^ Th and Th17 cells down in MLDSTZ treated mice, suggesting that phenotypically shifted Treg cells were not sufficiently suppressive.

### IL-2 production is not defective in MLDSTZ induced T1D

One possible explanation for the phenotypic shift of the Treg cells in our study could be that it is caused by a defective production of IL-2^20^. However, we did not find a defect in the IL-2 production by lymphocytes, CD4^+^CD25^−^ or CD8^+^ T cells in thymus, PDLNs and spleen of MLDSTZ treated mice (Supplementary Fig. 7A). We did observe that the proportion of Foxp3^−^Helios^+^IFN-γ^+^ T cells were increased on day 3 in the thymic glands of MLDSTZ treated mice (data not shown). These results suggest a decreased apoptotic stability depending on the Foxp3 transcription^20^. Therefore, the expression of Bcl-2 in Foxp3^+^ Treg cells was examined^20^,^21^. The Bcl-2 expression was not altered in Treg cells of thymus and spleen, but was decreased in PDLNs of MLDSTZ treated mice on day 21 (Supplementary Fig. 7B). Our results indicate that there was no impairment of the IL-2 production in MLDSTZ treated mice, and that the Foxp3^+^ Treg cells were apoptotically stable in thymus and spleen, but not in PDLNs.

### IL-35 administration both prevents and reverses MLDSTZ induced T1D

To further elucidate the role of IL-35 in early development of T1D, recombinant mouse IL-35 was administered to MLDSTZ treated mice as described in the Methods section.

**Figure 5:**
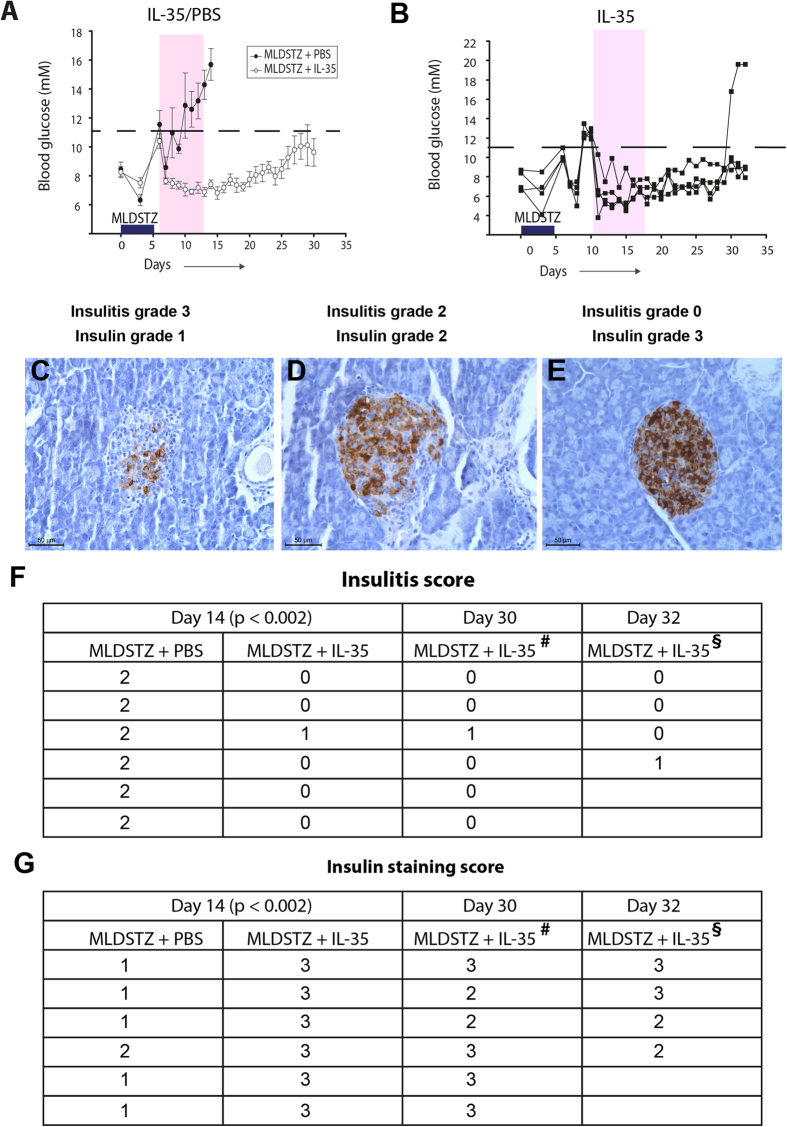
IL-35 prevents induction of MLDSTZ induced T1D and reverses established MLDSTZ induced T1D. (**A**) MLDSTZ treated mice were injected with mouse recombinant IL-35 (0.75 μg/day, i.p.) or 200 μl PBS/day i.p. from day 6 for 8 days (shaded area). Six mice from the control group and six mice from the IL-35 treated group were sacrificed on day 14. After discontinuing IL-35 treatment, one mouse became hyperglycemic on day 26, while five mice remained normoglycemic. (**B**) MLDSTZ treated mice that had been hyperglycemic for two consecutive days (on day 10, after the first injection of STZ) were treated with mouse recombinant IL-35 for 8 days (shaded area) (n = 4 mice). Representative images of (**C**) insulitis grade 3 (>2/3 of the islet area infiltrated with mononuclear cells) and insulin grade 1 (<25% of insulin-positive cells). (**D**) Insulitis grade 2 (1/3-2/3 of the islet area infiltrated with mononuclear cells) and insulin grade 2 (25–50% insulin-positive cells). (**E**) Insulitis grade 0 (no infiltration in islets) and insulin grade 3 (>75% insulin-positive cells). (**F**) Haematoxylin and eosin stained sections of pancreata of IL-35 (0.75 μg daily, i.p.) or PBS (200 μl, i.p.) treated mice were analyzed for insulitis by scoring (0, 1, 2, 3 and 4) as described in the Methods section. (**G**) Insulin stained sections of pancreata of MLDSTZ + PBS or MLDSTZ + IL-35 treated mice were analyzed for insulin by scoring (0, 1, 2, and 3) as described in the Methods section. ^**#**^MLDSTZ treated mice received IL-35 (0.75 μg daily, i.p.) for 8 days (starting from day 6 after the first injection of STZ). From day 14 (after the first injection of STZ) the IL-35 treatment was discontinued and mice were sacrificed on day 30 to analyze the morphology of pancreata. ^**§**^MLDSTZ treated mice that had been hyperglycemic for two consecutive days (on day 10) were treated with mouse recombinant IL-35 (0.75 μg daily, i.p.) for 8 days as indicated in Fig. 4B. The mice were sacrificed on day 32. Mann-Whitney Rank Sum tests were performed for comparisons between MLDSTZ + PBS and MLDSTZ + IL-35 treated groups.

Interestingly, MLDSTZ + IL-35 treated mice stayed normoglycemic during the treatment, whilst MLDSTZ + PBS treated mice became hyperglycemic from day 10 (Fig. 5A) and showed an increase in the degree of insulitis (Fig. 5C–F and Supplementary Fig. 8A). Five out of six MLDSTZ + IL-35 treated mice did not develop any insulitis at all (Fig. 5F and Supplementary Fig. 8A).

We also examined the effect of discontinuing the IL-35 treatment to investigate the long-term efficacy of IL-35 treatment. When IL-35 treatment was discontinued on day 14, five out of six mice remained normoglycemic until day 30 (Fig. 5A). The pancreatic islets of these mice displayed mild insulitis in some cases (Fig. 5F).

Finally, mice that had been hyperglycemic for two consecutive days (new onset diabetic) were treated with IL-35 for 8 days. These mice returned to normoglycemia after receiving the first dose of IL-35, and all but one mouse remained normoglycemic even after discontinuing the IL-35 treatment (Fig. 5B). The pancreata of these mice showed no or only mild insulitis (Fig. 5F). In addition, IL-35 treated mice had a higher score for insulin-positive staining and higher serum insulin concentration (Fig. 5G and Supplementary Fig. 8B). Moreover, the body weight of MLDSTZ + IL-35 treated mice was higher than that of MLDSTZ + PBS treated mice (Supplementary Fig. 8C). In conclusion, IL-35 administration prevented induction of, and reversed already established T1D by decreasing the insulitis and increasing the insulin content in pancreatic islets.

### IL-35 administration reversed the phenotypic shift of the Treg cells in MLDSTZ induced T1D

**Figure 6:**
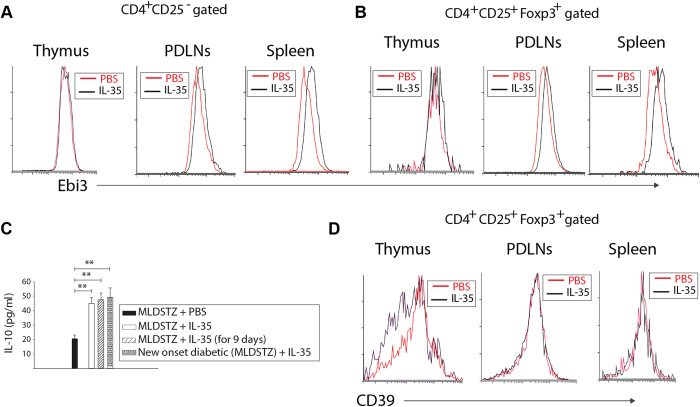
IL-35 administration increased the numbers of iT_R_35 cells and production of IL-10 in prevention of development of MLDSTZ induced T1D. Flow cytometry histograms showing the expression of Ebi3 in (**A**) CD4^+^CD25^−^ T cells or (**B**) CD4^+^CD25^+^Foxp3^+^ Treg cells of thymus, PDLNs cells and spleen of MLDSTZ + PBS or MLDSTZ + IL-35 treated mice on day 14. (**C**) The IL-10 concentration was determined in the serum of different groups of MLDSTZ + PBS and MLDSTZ + IL-35 treated mice by using ELSA assay. (**D**) Histograms showing the expression of CD39 in CD4^+^CD25^+^Foxp3^+^ Treg cells of thymus, PDLNs cells and spleen of MLDSTZ + PBS or MLDSTZ + IL-35 treated mice on day 14. Results are expressed as means ± SEM (n = 6), from two experiments (n = 3 mice/group/experiment). One-way ANOVA followed by Shapiro-Wilk tests (**C**) were performed for comparisons between IL-35 and PBS treated mice, ** denotes p < 0.01.

It has been reported that IL-35 suppresses autoimmunity by expanding the numbers of Treg cells^34^,^39^. However, in the present study we found that the proportions of Foxp3^+^ Treg and Foxp3^+^Helios^+^ tTreg cells were decreased in PDLNs and spleens of MLDSTZ + IL-35 treated mice, but the proportions of Foxp3^+^Helios^−^ pTreg cells did not differ between MLDSTZ + IL-35 and MLDSTZ + PBS treated mice (Supplementary Fig. 9A–C). The proportion of Foxp3^+^Nrp1^+^ tTreg were decreased, but the proportions of Foxp3^+^Nrp1^−^ pTreg cells was not altered in the PDLNs of MLDSTZ + IL-35 treated mice compared to MLDSTZ + PBS treated mice (Supplementary Fig. 9D–E), showing that the number of Treg cells was not increased by the IL-35 treatment. These might suggest that the IL-35 treatment has not affected the Treg cells. On the other hand, the increased MFI of Ebi3 in CD4^+^CD25^−^ T cells of PDLNs and spleen and Foxp3^+^ Treg cells of thymic glands, PDLNs and spleen of MLDSTZ + IL-35 treated mice (Fig. 6A and B, p < 0.05, not indicated in the figures), suggest an effect of IL-35 treatment on both Th and Treg cells. Furthermore, the serum IL-10 concentration was increased in IL-35 treated mice (Fig. 6C). Altogether, these results indicate that IL-35 administration in our model did not increase the numbers of Treg cells, but enhanced the production of anti-inflammatory cytokines (IL-10 and IL-35), which suppressed the development of MLDSTZ induced T1D.

Next, we determined the MFI of CD39 since Kochetkova *et al*. have reported that IL-35 administration stimulates the expression of CD39 on Treg cells in order to inhibit the development of collagen induced arthritis^40^. In the present study, we found that the expression of CD39 was not increased in thymus, PDLNs and spleen of MLDSTZ + IL-35 treated mice (Fig. 6D).

**Figure 7:**
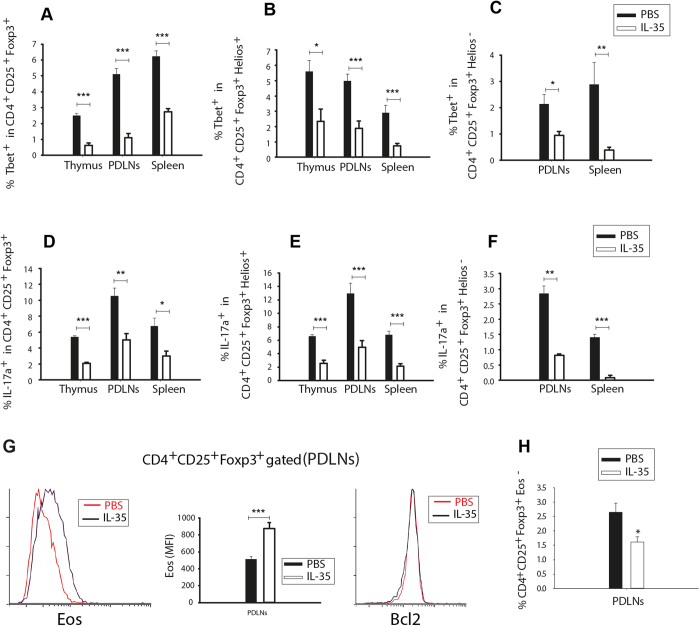
IL-35 administration reverses the phenotypic shift of the Treg cells in MLDSTZ induced T1D. The proportions of Tbet^+^ cells in (**A**) CD4^+^CD25^+^Foxp3^+^, (**B**) CD4^+^CD25^+^Foxp3^+^Helios^+^, and in (**C**) CD4^+^CD25^+^Foxp3^+^Helios^−^ Treg cells were analyzed in thymus, PDLNs, and spleen of MLDSTZ + PBS or MLDSTZ + IL-35 treated mice on day 14 by flow cytometry. (**D**–**F**) The proportions of IL-17a^+^ cells in (**D**) CD4^+^CD25^+^Foxp3^+^, (**E**) CD4^+^CD25^+^Foxp3^+^Helios^+^ and in (**F**) CD4^+^CD25^+^Foxp3^+^Helios^−^ Treg cells were analyzed by flow cytometry in thymus, PDLNs, and spleen of MLDSTZ + PBS or MLDSTZ + IL-35 treated mice on day 14. (**G**) Representative histograms are showing the expression of Eos (left panel), mean fluorescent intensity (MFI) of Eos in CD4^+^CD25^+^Foxp3^+^ Treg cells (middle panel), and Bcl-2 (right panel) in CD4^+^CD25^+^Foxp3^+^ Treg cells of PDLNs of MLDSTZ + IL-35 and MLDSTZ + PBS treated mice. (**H**) CD4^+^CD25^+^Foxp3^+^Eos^−^ Treg cells were analyzed in PDLNs of MLDSTZ + PBS or MLDSTZ + IL-35 treated mice on day 14 by flow cytometry. Results are expressed as means ± SEM, from two experiments (n = 3 mice/group/experiment). Unpaired t-tests were performed for comparisons between IL-35 and PBS treated mice and *, ** and *** denote p < 0.05, p < 0.01and p < 0.001, respectively.

To further investigate the effect or role of IL-35 in the early development of MLDSTZ T1D, we examined the numbers of Tbet^+^ and IL-17^+^ cells among Foxp3^+^ Treg cells, Foxp3^+^Helios^+^ tTreg cells, and Foxp3^+^Helios^−^ pTreg cells. Indeed, the numbers of Tbet^+^ and IL-17^+^ cells were decreased among the Treg cells of MLDSTZ + IL-35 treated mice (Fig. 7A–F). We also determined the numbers of IFN-γ^+^ cells in Foxp3^+^ Treg cells and found similar results as for Tbet (data not shown). The Eos expression was increased in Foxp3^+^ Treg cells of PDLNs of MLDSTZ + IL-35 treated mice, but the Bcl-2 expression was impaired (Fig. 7G). Furthermore, the proportion of Foxp3^+^Eos^−^ Treg cells was decreased in PDLNs of MLDSTZ + IL-35 mice (Fig. 7H). Thus, our results reveal that IL-35 administration enhances the expression of Eos in order to maintain the phenotype of Treg cells under MLDSTZ induced T1D conditions. In summary, these data indicate that IL-35 administration reverses the phenotypic shift of both tTreg and pTreg cells, thus protecting against MLDSTZ induced T1D.

Subsequently, we determined the numbers of leukocytes, Tc, Th and Th17 cells in IL-35 treated mice to investigate if the Treg cells are able to keep the numbers of these cells down. Bettini *et al*. have shown that the IL-35 suppresses autoimmune responses by blocking T cell proliferation^41^. In line with Bettini *et al*., in our study we found that numbers of leukocytes, Tc, and Th cells were decreased in PDLNs and spleen of MLDSTZ + IL-35 treated mice (Supplementary Fig. 10A–C). The numbers of Tbet^+^ or IL-17a^+^ cells among leukocytes, CD4^+^CD25^−^ T cells, CD8^+^ T cells were decreased in MLDSTZ + IL-35 treated mice (Supplementary Fig. 10D–G). Then, we investigated the proportion of IFN-γ^+^ cells among CD4^+^CD25^−^ T cells, CD8^+^ T cells and found similar results as for Tbet (data not shown). These results reveal that the Treg cells were functionally active in IL-35 treated mice.

The present results indicate a crucial role of IL-35 during early development of T1D in MLDSTZ. IL-35 administration prevented the development of T1D and reversed established T1D, maybe by recruiting more iT_R_35 cells and increasing the expression of Eos in Treg cells to maintain the regulatory T cell phenotype.

### IL-35 administration reverses established T1D in the NOD mouse model

**Figure 8:**
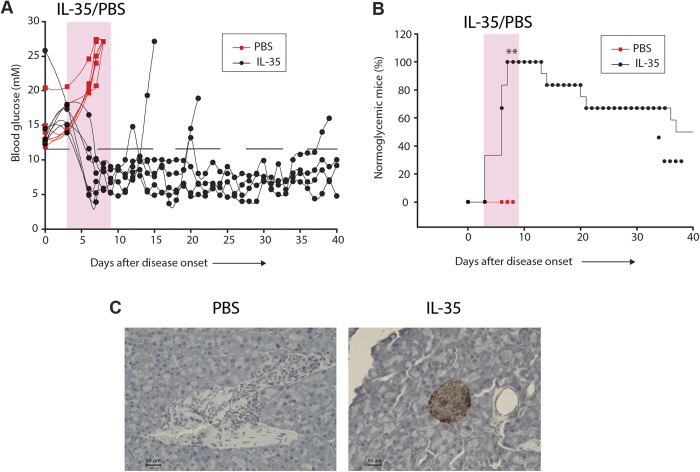
IL-35 administration reverses already established T1D in the NOD mouse model. (**A**) NOD mice that had been hyperglycemic (>11.1 mM) for two consecutive days (new-onset diabetic) were treated with PBS (red) or mouse recombinant IL-35 (0.75 μg daily, i.p.) (black) for 8 days as indicated. Blood glucose levels were followed for one month. (**B**) The percentage of diabetic mice remaining normoglycemic after the treatment with PBS or IL-35, ** denotes p = 0.002. Fisher’s exact test was performed for comparisons on day 5. (**C**) Representative images of the islets of PBS or IL-35 treated NOD mice. Results are expressed as means ± SEM (n = 6).

We subsequently investigated if the effect of IL-35 in counteracting T1D was confined to one murine T1D model. Therefore, we examined the NOD mouse model, a genetic mouse model of spontaneous T1D^42^. The proportions of Foxp3^+^ Treg, Helios^+^ tTreg, and Helios^−^ pTreg cells were increased in PDLNs of pre-diabetic (13-15 weeks old) NOD female mice compared to age matched wild type CD-1 mice (Supplementary Fig. 11A–C). The proportions of IFN-γ^+^ cells in Foxp3^+^ Treg, Helios^+^ tTreg, and Helios^−^ pTreg cells were increased in the PDLNs of NOD mice (Supplementary Fig. 11D–F). The degree of insulitis and the numbers of Foxp3^+^ cells were increased in pre-diabetic NOD mice compared to CD-1 mice (Supplementary Table 2). Thus, these data show that the Treg cell response in the NOD mouse is in line with our MLDSTZ experiments, suggesting a similar immunopathogenic mechanism of T1D development. To follow up on these findings, we treated recent onset diabetic NOD mice for 8 days with IL-35 or PBS. All the IL-35 treated mice reversed their diabetes after the first or second dose of IL-35, but the PBS treated mice remained diabetic (Fig. 8A,B). Furthermore, some mice remained normoglycemic even after discontinuing the IL-35 treatment. However, three out of six IL-35 treated NOD mice reverted to diabetes on days 11, 18, and 34 after disease onset (Fig. 8B). On the other hand, three out of six IL-35 treated diabetic mice remained normoglycemic until day 40. IL-35 treated NOD mice showed a higher score for insulin-positive staining grade and lower degree of insulitis compared to PBS treated NOD mice (Fig. 8C and Supplementary Table 3). Taken together, these data further confirmed that IL-35 treatment reverses established T1D, possibly by maintaining the Treg cell phenotype under autoimmune conditions.

### IL-35 treatment maintains the regulatory phenotype of Treg cells *in vitro*

**Figure 9:**
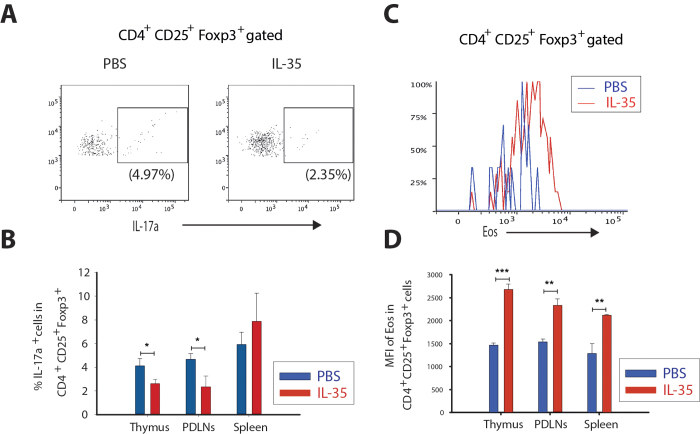
IL-35 treatment maintains the phenotype of Treg cells *in vitro*. (**A**) Thymocyte, PDLN cells and splenocytes were first gated for CD4, CD25 and Foxp3 expression, and then CD4^+^CD25^+^Foxp3^+^ Treg cells were further gated for IL-17a expression. Dot plots show representative experiments of PBS and IL-35 treated PDLNs cells of diabetic NOD mice. (**B**) The percentage of IL-17a^+^ cells in Foxp3^+^ Treg cells of thymic glands, PDLNs and spleen of PBS and IL-35 treated. (**C**) Thymocyte, PDLN cells and splenocytes were first gated for CD4, CD25 and Foxp3 expression, and then CD4^+^CD25^+^Foxp3^+^ Treg cells were further gated for Eos expression. Histogram shows representative experiments of PBS and IL-35 treated PDLNs cells of NOD diabetic mice. (**D**) The MFI of Eos in Foxp3^+^ Treg cells of thymic glands, PDLNs and spleen of PBS and IL-35 treated. Results are expressed as means ± SEM, from two experiments (n = 3 mice/group/experiment). Unpaired t-tests for comparisons between IL-35 and PBS treated groups were performed and *, ** and *** denote p < 0.05, p < 0.01and p < 0.001, respectively.

To further examine whether IL-35 maintains the regulatory phenotype of Treg cells in NOD mice. We isolated thymocytes, PDLN cells and splenocytes from severely diabetic NOD mice (>27.1 mM blood glucose) and treated the cells with IL-35 (10 ng/ml) overnight as described in the Methods section. IL-35 treatment decreased the expression of IL-17 and increased the Eos expression, thus effectively maintaining the phenotype, of thymic and PDLN Treg cells *in vitro*, but failed to maintain the phenotype of spleen Treg cells (Fig. 9A,B). In addition, the MFI of Eos was higher in IL-35 treated Treg cells than PBS treated Treg cells (Fig. 9C,D).

Our results further support that IL-35 reverses established T1D in NOD mice by maintaining the Treg cells phenotype, possible by inducing the expression of Eos, similar to what was seen in the MLDSTZ induced T1D model.

### Decreased circulating levels of IL-35 in humans with T1D

**Figure 10:**
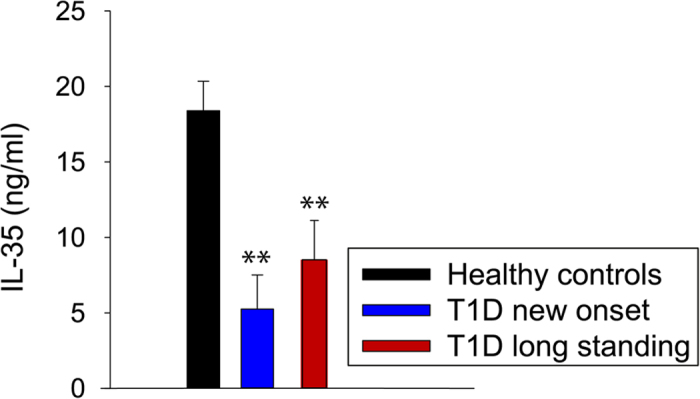
The circulating level of IL-35 is decreased in both new onset and long standing human T1D. Circulating IL-35 levels in healthy controls (n = 13), recent onset T1D (n = 8) and long-standing human T1D patients (n = 19). One-way ANOVA followed by Tukey’s test was performed for comparisons and **, denote p < 0.01.

IL-35 plasma levels were decreased in both recent onset (<1 year) and longstanding (1-5 years) human T1D patients compared to age-matched healthy controls (Fig. 10). Thus, our results indicate that IL-35 production may be impaired in human T1D patients as well.

## Discussion

We have found that both tTreg and pTreg cells are upregulated during the early development of experimental T1D, but this upregulation could not protect against hyperglycemia. This might be due to a phenotypic shift of the Treg cells and impaired production of IL-35. Furthermore, we found that IL-35 administration prevented the development of T1D and even reversed established T1D in two different mouse models. Moreover, IL-35 administration prevented β-cell destruction. The beneficial effects of IL-35 administration could be due to a reversal of the phenotypic shift of the Treg cells observed in diabetic animals, and by the promotion of an additional formation of iT_R_35 cells, as well as the induction of Eos expression in Treg cells. Hitherto, IL-35 has been known for enhancing the numbers of Treg^33^,^34^ and regulatory B (Breg) cells^43^,^44^ to suppress autoimmune and inflammatory responses, but herein we have found that IL-35 also maintains the phenotype of both tTreg and pTreg cells in autoimmune conditions. Furthermore, we found that the plasma levels of IL-35 were decreased in both recent onset and longstanding human T1D patients compared to healthy controls.

**Figure 11:**
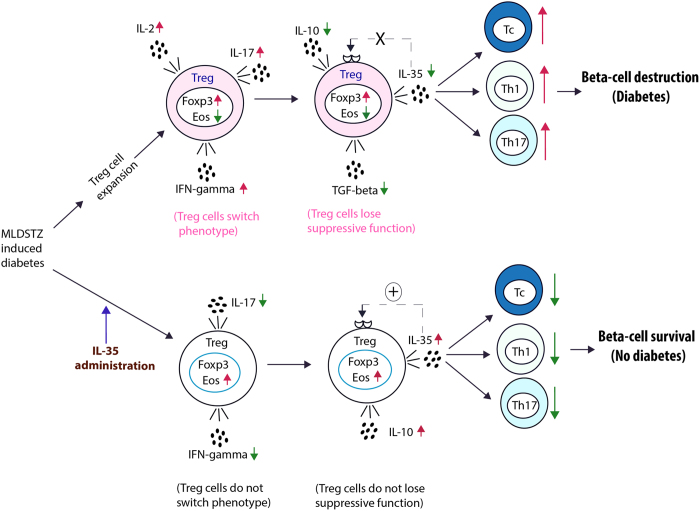
Tentative outline of IL-35 mediated protection against T1D in MLDSTZ. In MLDSTZ treated mice Treg cells are increased in numbers, but these Treg cells do not protect against hyperglycemia. This might be due to a phenotypic shift of the Treg cells leading to expression of IFN-γ, IL-2, and IL-17. The acquisition of a Th1 and Th17 like phenotype by the Treg cells may be caused by an impaired expression of Eos. This further leads to a decreased production of anti-inflammatory cytokines (IL-10, IL-35, and TGF-β) and a failure to keep the numbers of cytotoxic CD8^+^ (Tc), Th1 and Th17 cells down and ultimately β-cell destruction. IL-35 administration reversed the phenotypic shift of the Treg cells, possibly by inducing the expression of Eos and maintained the regulatory phenotype of Treg cells to both prevented from development, and reversed established T1D.

The increased numbers of Treg cells did not protect from diabetes development in either the MLDSTZ or the NOD mouse model. This could be due to a phenotypic shift and/or a functional defect of Treg cells in the early development of T1D. This notion was supported by our observation of an increase in the percentage of IFN-γ^+^ cells among tTreg and pTreg cells of MLDSTZ treated mice. Also, the impaired expression of Eos on days 10 and 21 is in agreement with the concept that Eos together with Foxp3 maintains the suppressive phenotype of Treg cells^37^. Moreover, the increased expression of IL-2 and IL-17 in Foxp3^+^ Treg cells confirmed that these cells had undergone a phenotypic shift. One could argue that IFN-γ, IL-2 or IL-17 expressing Foxp3^+^ Treg cells could still be phenotypically suppressive Treg cells, but our findings that the mRNA expression of IL-10, IL-35, and TGF-β were impaired and that the production of IL-10, IL-35, and TGF-β were decreased by CD4^+^CD25^+^ Treg cells, as well as that the numbers of Th and Th17 cells were increased in MLDSTZ treated mice even though the numbers of Treg cells were increased, suggest a functional impairment of the Treg cells. These results indicate that there is an insufficient production of anti-inflammatory cytokines due to a phenotypic shift of the Treg cells in autoimmune diabetes. This could lead to an inability to block the differentiation of Th17 cells, an increase in the Th1 cell numbers and decreased formation of iT_R_35 cells in MLDSTZ induced T1D^34^,^39^ (Fig. 11 and Supplementary Fig. 12). The observation of decreased circulating IL-35 concentrations in human T1D compared to healthy controls indicates that this model might also be applicable to the human immune system.

The stability of Foxp3 has been debated, but according to our findings, the Foxp3 mRNA expression is stable under our experimental conditions. However, we also observed a decreased expression of Bcl-2 in Foxp3^+^ Treg cells in PDLNs, but not in the thymus and spleen, on day 21. This indicates that the Foxp3 gene is unstable under these conditions in PDLNs. Tang *et al*. have shown an instability of the Foxp3 gene in pancreatic islets of diabetic mice, that was due to insufficient production of IL-2^20^. Our results indicate that the IL-2 production was not defective in MLDSTZ induced T1D, similar to what has been observed in human T1D^45^, thus indicating that the Treg cells observed in our study are mostly stable. Sharma *et al*. have shown that Foxp3^+^Eos^−^ Treg cells are functional Treg cells that have a tendency to change their phenotype into Th1 or Th17 cells under inflammatory conditions. These cells are further characterized as Foxp3^+^Eos^−^CD38^+^ and this subset of Treg cells is stable^37^. In our model, both Foxp3^+^Eos^−^ and Foxp3^+^Eos^−^CD38^+^ Treg cells were increased as the disease was progressing. In line with our findings concerning Eos, Lempainen *et al*. have recently shown an inverse correlation of the *IKZF4* gene, that encodes Eos, with insulin autoantibodies in T1D patients early after diagnosis^46^, suggesting a role for Eos in the development of T1D.

Exogenous administration of IL-35 effectively prevented T1D development and reversed already established T1D in both MLDSTZ mice and NOD mice. This could be caused by a reversal of the phenotype of T cells (from Th1 or Th17 to suppressive Treg) and/or by increasing the expression of Eos in Treg cells (Fig. 11). Another possible explanation is that external IL-35 administration may recruit more iT_R_35 cells, and increase production of both IL-10 and IL-35 by the Treg cells (Supplementary Fig. 12). This notion was further supported when we observed a higher concentration of serum IL-10 in MLDSTZ + IL-35 mice. The impaired expression of CD39 in Foxp3^+^ Treg cells of MLDSTZ + IL-35 treated mice further support this hypothesis, since IL-35 has been shown to induce CD39 expression in order to dampen arthritis by inducing Treg cells^40^. The impaired expression of Bcl-2 and CD39, in combination with the increased expression of Eos in Treg cells of MLDSTZ + IL-35 treated mice suggest that IL-35 may play a role in maintaining the Treg cell phenotype in autoimmune conditions by the induction of expression of Eos in Treg cells. Moreover, decreased proportions of Foxp3^+^Eos^−^ Treg cells were observed in MLDSTZ + IL-35 mice. In addition, IL-35 maintained the phenotype of Treg cells *in vitro* by inducing Eos expression.

Although IL-35 administration did not increase the number of Treg cells, it decreased the number of Th1, Th17 cells and IFN-γ or IL-17a expressing CD8^+^ T cells, and reduced the infiltration of mononuclear cells in the islets. In line with our findings, Bettini *et al*. have also shown that ectopic expression of IL-35 in β-cells of NOD mice did not increase the number of Treg cells, but that the ectopic expression of IL-35 prevented T1D development^41^. In addition, our data illustrate that IL-35 may play a role in maintaining the phenotype of Treg cells to both prevent development of, and to reverse established T1D.

It has been reported that Nrp1 could have a role in T1D^47^, diabetic retinopathy^48^ and diabetic nephropathy^49^. Interestingly, in our study IL-35 administration reduced the numbers of Foxp3^+^Nrp1^+^ cells, which may suggest a protective role of IL-35 also in other aspects of diabetes. Furthermore, higher insulin positive scores in the islets of either MLDSTZ + IL-35 or IL-35 treated NOD mice indicate a protective and/or regenerative effect of IL-35 on insulin producing β-cells. The present study described the role of IL-35 in early development of T1D and suggests that IL-35 could be used to treat human T1D and possibly also other autoimmune disorders.

Growing evidence is suggesting that in most autoimmune diseases, Foxp3^+^ Treg cells lose their suppressive phenotype, which leads to an increase in the number and function of Th1 or Th17 cells, causing disease development^23^,^35^. Here, we provide the first preclinical results that IL-35 administration can prevent induction of, and reverse established murine T1D possibly by affecting the phenotypic properties of Treg cells. Together with the observation that ectopic expression of IL-35 in β-cells can prevent T1D in NOD mice^41^, our data indicate that IL-35 could be used as a therapeutic target not only for T1D, but perhaps also for other autoimmune, inflammatory and infectious diseases. That decreased concentration of circulating IL-35 was found in human T1D patients compared to healthy controls, implies an exciting potential for IL-35 as a possible treatment in human T1D, perhaps in both recent onset and established T1D. In line with our hypothesis, several other groups have recently reported that IL-35 play a role in other autoimmune and inflammatory diseases^50^,^51^,^52^,^53^,^54^,^55^,^56^. Beside the putative mechanism for IL-35 induced protection against T1D outlined herein (Fig. 11 and Supplementary Fig. 12), it cannot be excluded that IL-35 may possess other yet unknown immunosuppressive properties as well.

## Methods

### Mice and MLDSTZ *in vivo* treatment

The local animal ethics committee at Uppsala University approved the animal experiments. Male CD-1 mice aged 7–15 weeks and weighing 28–36 g were used. The mice were obtained from Charles River (Hannover, Germany). The animals were used accordance with international guidelines (NIH publications 85-23). Male CD-1 Mice were injected intraperitoneally (i.p.) with streptozotocin (STZ) (Sigma, St Louis, MO, USA; 40 mg/kg body weight) dissolved in saline or 200 μl of saline (vehicle) for 5 consecutive days, starting on day 0^27^,^57^. Untreated mice (n = 7) constituted a naïve control group. The experiments were performed twice with three mice in each experimental group, giving a total of six mice in each experimental group. Blood glucose concentrations were measured 0, 3, 7, 10, 14 and 21 days after the first injection of STZ, using a blood glucose meter (Medisense, London, UK). Blood samples were obtained from the tail vein of non-fasted mice. Blood glucose levels above 11.1 mM were considered hyperglycemic.

The mice were weighed before MLDSTZ or saline injections and when sacrificed on days 3, 7, 10, 14 and 21. The naïve control group of mice was sacrificed on day 0. A piece of pancreas (approximately 1/10) was immediately removed, flash-frozen in liquid nitrogen and stored at −80 °C until RNA isolation. The remaining part of the pancreas was transferred to 10% formalin for morphological analysis. The spleen was also removed and a small piece (approximately 1/3) of the spleen was fixed in 10% formalin. The remaining spleen was placed on ice in Hanks’ balanced salt solution (Sigma, Stockholm, Sweden) supplemented with antibiotics. The thymic glands were removed and placed on ice in Hanks’ balanced salt solution. Pancreatic draining lymph nodes (PDLNs) were removed and placed on ice in RPMI1640 medium (Sigma Aldrich, St Louis, MO, USA) supplemented with antibiotics.

The NOD mice used were originally obtained from the Clea Company (Aobadi, Japan), and have subsequently been inbred and kept under pathogen-free conditions at the animal department, Biomedical Center, Uppsala University, Uppsala, Sweden.

### Cell isolation from thymic glands, PDLNs and spleen

Single cell suspensions of thymic glands, PDLNs and spleen tissue were made as previously described^32^,^58^. The cell suspensions were counted using a FACSCalibur (BD, Franklin Lakes, NJ, USA) and 10^6^ cells were stained for flow cytometry analysis. The remaining cells were transferred into RLT buffer (RNeasy Plus Mini kit, Qiagen, Hilden, Germany) containing 1% β-mercaptoethanol and stored at −80 °C until RNA extraction.

### Flow cytometry staining

Cells were stained for Foxp3 flow cytometry analysis according to the staining procedure described in the Mouse Regulatory T Cell Staining Kit # 3 (eBioscience, San Diego, CA, USA). We did not stimulate the cells prior to cytokine staining since it could have caused artefacts upon Foxp3 staining^37^.

Antibodies from eBioscience were as follows: anti-CD4 (RM4-5), anti-CD25 (PC61.5), anti-CD38 (90), anti-CD39 (24DMS1), anti-CD44 (IM7), anti-Eos (ESB7C2) and anti-Foxp3 (FJK-16s). The following antibodies were from Biolegend, San Diego, USA: anti-Bcl2 (BCL/10C4), anti-Helios (22F6), anti-IFN-γ (XMG1.2), anti-IL-2 (JES6-5H4), anti-IL-17 (TC11-18H10.1) and anti-T-bet (4B10). The anti-CD8 (53-6.7) was from BD and anti-Nrp1 (761705), anti-Ebi3 (355022) and anti-IL-12p35 (27537) were from R&D, Minneapolis, MN, USA.

The stained cells were analyzed on a FACSCalibur or BD LSRII Flow Cytometry (BD) at the core facility (BioVis) Uppsala University, Uppsala, Sweden. The data were analyzed with Diva 6.0 software (BD) or Flowlogic software (Inivai Technologies, Australia). Gating strategies were made using single stained and fluorescence minus one-stained controls. Manufacturer’s instructions (BD) were followed strictly while analyzing the data and performing the flow cytometry staining.

Note: When we refer to CD4^+^ in thymic glands in this paper, it means CD4^+^CD8^−^ and CD8^+^ means CD8^+^CD4^−^.

### Morphological analysis of pancreas

Paraffin embedded pancreas were sectioned at 5–7 μm thickness. In between each section, 5-6 sections were discarded to cover the entire tissue area and to avoid including the same cells in consecutive sections. In total 5 slides containing 4-5 tissue sections on each slide were prepared from each mouse for haematoxylin and eosin staining. The slides were analyzed in a blinded manner under a light microscope. The degree of insulitis was graded as 1, 2, 3 and 4 as described previously^42^.

### Histological analysis of Foxp3^+^ cells in pancreas and spleen

The Foxp3 staining was performed as described previously^58^.

Foxp3 stained tissue sections were analyzed with a DAS Mikroskop LEITZ DMR microscope. The pancreatic tissue sections were divided into exocrine tissue, islets of Langerhans (or endocrine tissue), stroma and pancreatic lymph nodes (PLNs), and these areas were analyzed separately. PLNs were not included in the final analysis, since we found very few in the sections. The following arbitrary scoring system was used; Endocrine tissue: 0; no infiltrating Foxp3^+^ cell, 1; 1-3 infiltrating Foxp3^+^ cells, 2; 3-10 infiltrating Foxp3^+^ cells and 3; >10 infiltrating Foxp3^+^ cells (Supplementary Figure 2A–D). Exocrine tissue: 0; no Foxp3^+^ cell, 1; 1-3 Foxp3^+^ cells and 2; >3 Foxp3^+^ cells (Supplementary Figure 1E–F). Stroma: 0; no Foxp3^+^ cell and 1; >1 Foxp3^+^ cells (data not shown). Five slides from each mouse, containing 4-5 tissue sections, were analyzed blindly and median values were calculated for statistical analysis.

Four images from each Foxp3 stained tissue section were taken using a light microscope (at x100 magnification). In total, 20 images were taken from each spleen. The images were analyzed semi quantitatively and blindly using the ImageJ software (Version 1.45s. downloaded from http://http://imagej.nih.gov/ij). The Foxp3^+^ cells in the red and white pulp were counted manually.

### Histological analysis of Ebi3^+^ cells among Foxp3^+^ cells in pancreatic tissue

Ten consecutive sections from paraffin embedded pancreatic tissues were made. Among these sections five alternative sections were stained for Foxp3 as described earlier and remaining sections were stained for Ebi3. Ebi3 staining was made as follow: (1) The antigen retrieval was done by using Diva Decloaker buffer (BIOCARE MEDICAL) or Tris Buffered Saline buffer, pH 9.0. (2) Tissue sections were treated with 10% hydrogen peroxide to remove endogenous peroxidase. (3) Swine serum (1:20) was used to block the tissue sections (30 minutes). (4) Tissue sections were incubated overnight with anti-human/mouse Ebi3 (20 μg/ml; Novus Biologicals, Littleton, Colorado, USA) in a humidified chamber at 4 °C. (5) The next day, HRP polyclonal swine anti-rabbit antibody (1:200; Dako AB, Stockholm, Sweden) was applied for 60 minutes then visualized with DAB substrate (Sigma–Aldrich Sweden AB). (6) The sections were counterstained with Mayer’s hematoxylin (Histolab, Gothenburg, Sweden). Spleen tissue sections were used as positive controls.

Consecutive sections stained for Ebi3 or Foxp3 were analyzed using a Leica’s light microscope.

### Insulin staining of pancreatic tissues

The insulin staining and image analysis was performed as described previously^58^.

### Quantitative RT-PCR

Total RNA was extracted from PDLNs and spleen cells using the RNeasy Plus Mini kit (Qiagen, Hilden, Germany) following the manufacturer’s instructions. To isolate total RNA from pancreas, the RNeasy Mini kit (Qiagen) was used to improve the yield. cDNA was made from RNA using a reverse transcriptase kit (QuantiTect Reverse Transcription, Qiagen) and random primers supplied by the manufacturer.

Real-Time PCR was performed for detection of Foxp3 in PDLNs, spleen and pancreas cDNA, and using β-actin as the housekeeping gene. The RT-PCR on pancreas and PDLN cells was run on the Light Cycler instrument (Light Cycler 2.0, Roche, Basel, Switzerland) using the Light cycler Fast Start DNA Master Hybridization Probes Kit (Roche). Quantitative RT-PCR analysis for Foxp3 expression in spleen cDNA and Ebi3, IL-12p35, IL-10, TGF-β, IL-17 and β-actin in PDLN, pancreas and spleen cDNA was performed and analyzed as previously described^58^. All the primers (Supplementary Table 4) and hybridization probes were designed by and obtained from TIB Molbiol Syntheselabor (Berlin, Germany).

The following hybridization probes were used:

Foxp3 probes:

5′-CCATTGGTTTACTCGCATGTTCGCC—FL LC640-

ACTTCAGAAACCACCCCGCCACCT—PH

β-actin probes:

5′-TCTCCCTCACGCCATCCTGCGTCT—FL 5′-LC705-

ACCTGGCTGGCCGGGACCTGA—PH

### IL-35 administration

Mouse recombinant IL-35 (Chimerigen, Liestal, Switzerland) was administered i.p. (0.75 μg/day, dissolved in 200 μl PBS) to the mice. The control group received 200 μl PBS/day and blood glucose concentrations were monitored daily. Four different protocols were employed to study the effects of IL-35 administration:

(1) The mice received STZ (40 mg/kg body weight) for the first five consecutive days (MLDSTZ), followed from day 6 by IL-35 or PBS i.p. administration for 8 days, and then the mice were killed on day 14. The organs (thymic glands, PDLNs, spleen and pancreas) were removed as described for flow cytometry or morphological analysis above.

(2) MLDSTZ treated mice were treated with IL-35 from day 6 for 8 days. The IL-35 treatment was discontinued from day 14 and the mice were killed on day 30. Their pancreases were removed for morphological analysis.

(3) MLDSTZ treated mice did not receive any IL-35 until the mice had become diabetic (blood glucose >11.1 mM). Diabetic mice received IL-35 for 8 days, and then the treatment was discontinued and the animals were killed on day 32. Pancreases were removed for morphological analysis.

(4) Recent onset diabetic NOD mice were treated with mouse recombinant IL-35 for 8 days or PBS for 3 days. The mice that remained normoglycemic after IL-35 treatment were sacrificed on day 40. However, mice that reverted to diabetes after IL-35 treatment were sacrificed if they had hyperglycemia blood glucose levels (>11.1 mM) for two consecutive days. PBS treated NOD mice were sacrificed on day 5 by cervical dislocation.

### CD4^+^CD25^+^ Treg cells sorting

CD4^+^CD25^−^ T and CD4^+^CD25^+^ Treg cells from thymic glands, PDLNS and spleen were sorted by using the Miltenyi Biotec magnetic sorter (Miltenyi Biotec, Germeny) and the CD4^+^CD25^+^ Treg cells isolation kit (Miltenyi Biotec) as described previously^32^.

### *In vitro* cells stimulation

The cells were stimulated with plate bound anti-CD3 (2 μg/ml) and anti-CD28 (2 μg/ml) as described earlier^32^.

### *In vitro* IL-35 treatment

Single cells suspensions of thymic glands, PDLNs and spleen of NOD diabetic mice (>27.1 mM, blood glucose) were stimulated with plate bound anti-CD3 (2 μg/ml) and anti-CD28 (2 μg/ml) in 24-well-plates for overnight in the presence or absence of IL-35 (10 ng/ml). The next day, cells were harvested and stained for flow cytometry analysis.

### Enzyme-linked immunesorbent assay

Serum samples were analyzed in duplicate to determine the insulin (Mercodia, Uppsala, Sweden), IL-10 (R&D Systems), IL-35 (Biolegend) and TGF-β (Biolegend) concentrations in mice. The concentrations of IL-10, IL-35 and TGF-β were also determined in cell supernatants of stimulated CD4^+^CD25^+^ Treg cells (50 × 10^4^ cells/well) by using ELISA. Plasma IL-35 concentration in humans was determined using a IL-35 ELISA kit assay (Biolegend).

### Human plasma samples

This part was approved by the Uppsala County regional ethics board and carried out in accordance with the principles of the Declaration of Helsinki as revised in 2000. All participants were supplied with oral and written information and gave written consent prior to inclusion and informed consent was obtained from all the study participants. Descriptive data for healthy controls and patients with T1D are given in supplementary Table 5.

### Statistical analysis

The Sigmaplot 12.03 software was used for the statistical analysis. Unpaired t-tests were used for comparions between two groups. Mann-Whitney Rank Sum Tests were performed for nonparametric observations. Detailed information on what tests were used for the different experiments is included in the figure legends. To compare the number of Foxp3^+^ cells in pancreas of MLDSTZ with vehicle treated mice, the median values from day 7 to day 21 were considered as one group and then a Mann-Whitney Rank Sum Test was performed between the MLDSTZ and vehicle group. The results are expressed as means ± SEM. A p-value below 0.05 was considered statistically significant.

## Additional Information

**How to cite this article**: Singh, K. *et al*. Interleukin-35 administration counteracts established murine type 1 diabetes – possible involvement of regulatory T cells. *Sci. Rep*. **5**, 12633; doi: 10.1038/srep12633 (2015).

## Supplementary Material

Supplementary Information
