# Supplementary material for: Interleukin-35 administration counteracts established murine type 1 diabetes – possible involvement of regulatory T cells
Source: Sci Rep. 2015 Jul 30;5:12633. doi: 10.1038/srep12633 (PMC4519737; doi:10.1038/srep12633)
Supplement: Supplementary Information [file srep12633-s1.pdf]

Supplementary material

Interleukin-35 administration counteracts established murine type 1 diabetes – possible involvement of regulatory T cells

Kailash Singh*[1], Erik Kadesjö[1], Julia Lindroos[1], Marcus Hjort[1], Marcus Lundberg[1], Daniel Espes[1,2], Per-Ola Carlsson[1,2], Stellan Sandler[#1] and Lina Thorvaldson[#1]

[#]Shared authorship

[1]Department of Medical Cell Biology, Biomedical Centre, Uppsala University, Uppsala, Sweden.

[2]Department of Medical Sciences, Uppsala University, Uppsala, Sweden

***Corresponding author**

Kailash Singh, Department of Medical Cell Biology, Uppsala University

Box 571, Husargatan 3

75123 Uppsala, Sweden

email: Kailash.Singh@mcb.uu.se

Supplementary figure 1. The proportions of $CD4^+$ and $CD8^+$ Treg cells and the numbers of $Foxp3^+$ cells are increased in MLDSTZ induced T1D.

(A) Thymocyte, PDLN cells and splenocytes were first gated for CD4 and CD25 expression, and then $CD4^+CD25^+$ T cells were further gated for Foxp3 expression. Dot plots show representative experiments of vehicle and MLDSTZ injected mice thymocytes, PDLNs cells and splenocytes. (B) Thymocytes, PDLN cells and splenocytes were gated for CD8 and Foxp3, and then $CD8^+Foxp3^+$ Treg cells were analyzed. Dot plots show representative experiments from thymocytes, PDLNs cells and splenocytes from vehicle or MLDSTZ injected mice. (C) The numbers of $Foxp3^+$ cells in red pulp, white pulp and whole spleen were analyzed as described in the Methods section. (D) The proportion of $CD8^+Foxp3^+$ Treg cells in naïve, vehicle and MLDSTZ treated mice. Results are expressed as means ± SEM (n = 6), from two experiments (n = 3 mice/group/experiment). Unpaired t-tests were performed for comparisons between vehicle and MLDSTZ treated groups on corresponding days. ** and *** denote $p < 0.05$, $p < 0.01$, and $p < 0.001$, respectively.

Supplementary figure 2. Grading of $Foxp3^+$ staining in endocrine and exocrine pancreata of MLDSTZ induced T1D

(A) Representative images of grade 0: no $Foxp3^+$ cells per islet. (B) Grade 1: 1-3 $Foxp3^+$ cells per islet. (C) Grade 2: 3-10 $Foxp3^+$ cells per islet and (D) Grade 3: >10 $Foxp3^+$ cells per islet. (E) Grade 1: 1-3 $Foxp3^+$ cells in exocrine pancreas. (F) Grade 2: >3 $Foxp3^+$ cells in exocrine pancreas.

Supplementary figure 3. Both tTreg cells and pTreg cells are increased in MLDSTZ induced T1D.

(A-B) The proportions of $Foxp3^+Helios^+$ tTreg and $Foxp3^+Helios^-$ pTreg cells in thymocytes, PDLNs cells and splenocytes of vehicle or MLDSTZ treated mice. (C-D) The proportions of $Foxp3^+Nrp1^+$ tTreg and $Foxp3^+Nrp1^-$ pTreg cells in thymic glands, PDLNs and spleen of vehicle or MLDSTZ treated mice. Results are expressed as means ± SEM (n = 6), from two experiments (n = 3 mice/group/experiment). Unpaired t-tests were performed for comparisons between vehicle and MLDSTZ treated groups on corresponding days. *, ** and *** denote $p < 0.05$, $p < 0.01$, and $p < 0.001$, respectively.

Supplementary figure 4. Impaired expression of IL-35, IL-10, and TGF-β in MLDSTZ induced T1D.

(A-D) Relative (A) Ebi3, (B) IL-12p35, (C) IL-10 and (D) TGF-β mRNA expression in PDLN cells (on day 10) and splenocytes (on day 21) from vehicle or MLDSTZ injected mice were analyzed by real time RT-PCR. Results are expressed as means ± SEM (n = 6), from two experiments (n = 3 mice/group/experiment). (E) The MFI of IL-12p35 in $CD4^+CD25^+Foxp3^+$ Treg cells. (F) The MFI of Ebi3 in $CD4^+CD25^+Foxp3^+$ Treg cells. Unpaired t-tests were performed for comparisons between vehicle and MLDSTZ treated groups on corresponding days. *, ** and *** denote $p < 0.05$, $p < 0.01$, and $p < 0.001$, respectively.

Supplementary figure 5. Both tTreg and pTreg cells acquire a T effector cell phenotype in MLDSTZ induced T1D.

(A) Thymocytes, PDLN cells and splenocytes were gated for CD4, CD25 and Foxp3 expression, and then $CD4^+CD25^+Foxp3^+$ Treg cells were further gated for Foxp3 and IFN-γ expression. The percentage of IFN-γ^+ cells among $CD4^+CD25^+Foxp3^+$ Treg

cells were analyzed by flow cytometry. The The percentage of IFN-γ^+ cells in (B) $Foxp3^+Helios^+$ tTreg cells and (C) $Foxp3^+Helios^-$ pTreg cells of thymic glands, PDLNs and spleen of vehicle or MLDSTZ injected mice were analyzed by flow cytometry. Results are expressed as means ± SEM (n = 6), from two experiments (n = 3 mice/group/experiment). Unpaired t-tests or Mann-Whitney Rank Sum Tests (C; spleen) were performed for comparisons between vehicle and MLDSTZ treated mice. *, ** and *** denote $p < 0.05$, $p < 0.01$, and $p < 0.001$, respectively. ND: not detected.

Supplementary figure 6. Both $Foxp3^+Eos^-$ and $Foxp3^+Eos^-CD38^+$ Treg cells are increased in MLDSTZ induced T1D.

PDLN cells and splenocytes were gated for CD4, CD25 and Foxp3 expression, and then $CD4^+CD25^+Foxp3^+$ Treg cells were further gated for Foxp3 and Eos expression. $Foxp3^+Eos^-$ Treg cells were further gated for CD38 expression. Dot plots are representative of vehicle and MLDSTZ injected mice for PDLNs cells and splenocytes (upper panels). Results are expressed as means ± SEM (n = 6), from two experiments (n = 3 mice/group/experiment). (A) The proportions of $CD4^+CD25^+Foxp3^+Eos^-$ (B) and $CD4^+CD25^+Foxp3^+Eos^-CD38^+$ Treg cells in PDLNs and spleen of vehicle and MLDSTZ treated mice. Unpaired t-tests were performed for comparisons between vehicle and MLDSTZ treated mice. *, and ** denote $p < 0.05$, and $p < 0.01$, respectively.

Supplementary figure 7. IL-2 production in MLDSTZ induced T1D is not defective.

(A) Unstimulated leukocytes, $CD4^+CD25^-$ Th, or $CD8^+$ T cells were analyzed for expression of IL-2 in thymic glands, PDLNs or spleen of vehicle (blue) or MLDSTZ (red) treated mice. (B) Bcl-2 expressing $CD4^+CD25^+Foxp3^+$ Treg cells in thymus, PDLNs, and spleen of vehicle (blue) and MLDSTZ (red) treated mice were analyzed by flow cytometry on days 7 and 21. Histograms are representative of two independent experiments (n = 3 mice/group/experiment).

Supplementary figure 8. IL-35 administration prevents both insulitis and β-cell destruction.

(A) Representative images of the islets of MLDSTZ + PBS or MLDSTZ + IL-35 treated mice on day 14. (B) Insulin concentrations were analyzed in serum from MLDSTZ + PBS and MLDSTZ + IL-35 treated mice (as indicated in the figure) using ELISA. (C) Change in body weight, expressed as percentage of the baseline values. Results are expressed as means ± SEM (n = 6), from two experiments (n = 3 mice/group/experiment). One-way ANOVA followed by Shapiro-Wilk tests (B) and Unpaired t-tests (C) were performed for comparisons between IL-35 and PBS treated mice, * and ** denote $p < 0.05$, and $p < 0.01$, respectively.

Supplementary figure 9. IL-35 administration did not increase the numbers of Treg cells.

The proportions of (A) $CD4^+CD25^+Foxp3^+$ Treg, (B) $CD4^+CD25^+Foxp3^+Helios^+$ tTreg, and (C) $CD4^+CD25^+Foxp3^+Helios^-$ pTreg cells were analyzed in thymus, PDLNs, and spleen of MLDSTZ + PBS or MLDSTZ + IL-35 treated mice on day 14 by flow cytometry. The proportions of (D) $CD4^+CD25^+Foxp3^+Nrp1^+$ tTreg, (E) $CD4^+CD25^+Foxp3^+Nrp1^-$ pTreg cells were analyzed in PDLNs of MLDSTZ + PBS or MLDSTZ + IL-35 treated mice on day 14 by flow cytometry. Results are expressed as

means ± SEM (n = 6), from two experiments (n = 3 mice/group/experiment). Unpaired t-tests were performed for comparisons between IL-35 and PBS treated mice. *, and ** denote $p < 0.05$, and $p < 0.01$, respectively.

Supplementary figure 10. IL-35 administration kept the numbers of Th1 and Th17 cells down to prevent from development of MLDSTZ induced T1D.

The absolute numbers of (A) leukocytes, (B) $CD4^+CD25^-$ Th cells and (C) $CD8^+$ T cells were analyzed by flow cytometry in thymocytes, PDLNs cells and splenocytes from MLDSTZ + PBS or MLDSTZ + IL-35 treated mice on day 14. The proportions of $Tbet^+$ cells in (D) $CD4^+CD25^-$ Th or (E) $CD8^+$ T cells, and IL-$17a^+$ cells in (F) $CD4^+CD25^-$ Th or (G) $CD8^+$ T cells of thymus, PDLNs and spleen of MLDSTZ + PBS or MLDSTZ + IL-35 treated mice on day 14. Results are expressed as means ± SEM (n = 6), from two experiments (n = 3 mice/group/experiment). Unpaired t-tests were performed for comparisons between IL-35 and PBS treated mice. * and ** denote $p < 0.05$, and $p < 0.01$, respectively.

Supplementary figure 11. Treg cells are increased in NOD mouse model of T1D and Treg cells are phenotypically shifted in NOD mice.

The proportions of (A) $CD4^+CD25^+Foxp3^+$ Treg, (B) $CD4^+CD25^+Foxp3^+Helios^+$ tTreg, and (C) $CD4^+CD25^+Foxp3^+Helios^-$ pTreg cells in CD-1 or NOD mice were analyzed in PDLNs by flow cytometry. The proportions of IFN-γ^+ cells in (D) $CD4^+CD25^+Foxp3^+$ Treg, (E) $CD4^+CD25^+Foxp3^+Helios^+$ tTreg, and (F) $CD4^+CD25^+Foxp3^+Helios^-$ pTreg cells were analyzed in PDLNs of CD-1 or NOD treated mice on day 14 by flow cytometry. Results are expressed as means ± SEM,

from two experiments (n = 3 mice/group/experiment). Unpaired t-tests were performed for comparisons between IL-35 and PBS treated mice. *, and ** denote $p < 0.05$, and $p < 0.01$, respectively.

Supplementary figure 12. Schematic representation of the function of IL-35

IL-35 is produced by the $CD4^{+}CD25^{+}Foxp3^{+}$ Treg cells. IL-35 activate Tconv cell, which have receptors for IL-35, to further produce IL-35. This type of cell is characterized as $iT_{R}35$. IL-35 also has an auto-feedback regulation on Treg cells to induce a higher production of IL-10 and IL-35. IL-10 and IL-35 block the differentiation of Th17 cells and Th1 cells, resulting in a decreased production of pro-inflammatory cytokines.

Supplementary Figure 1

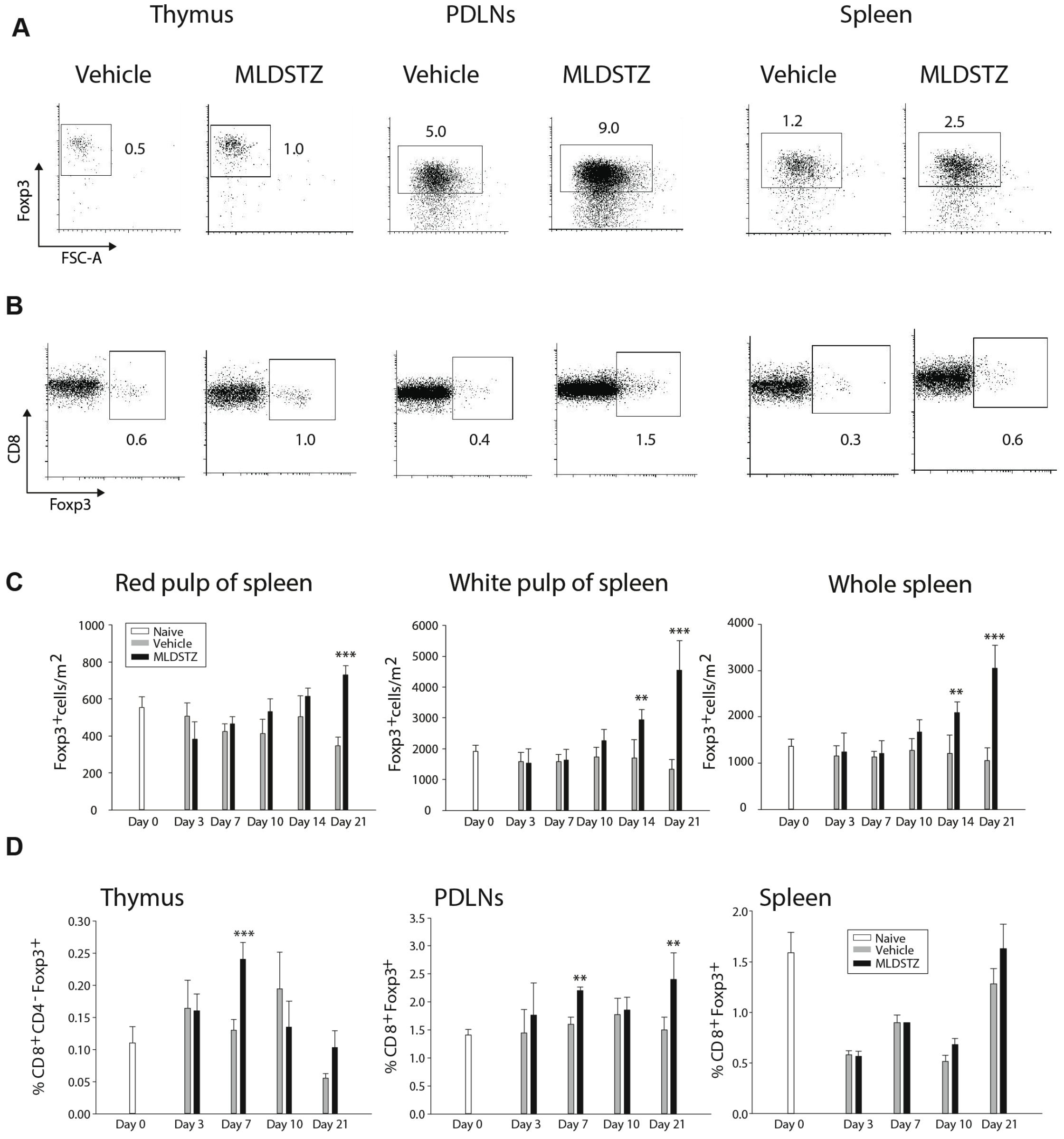

Supplementary Figure 2

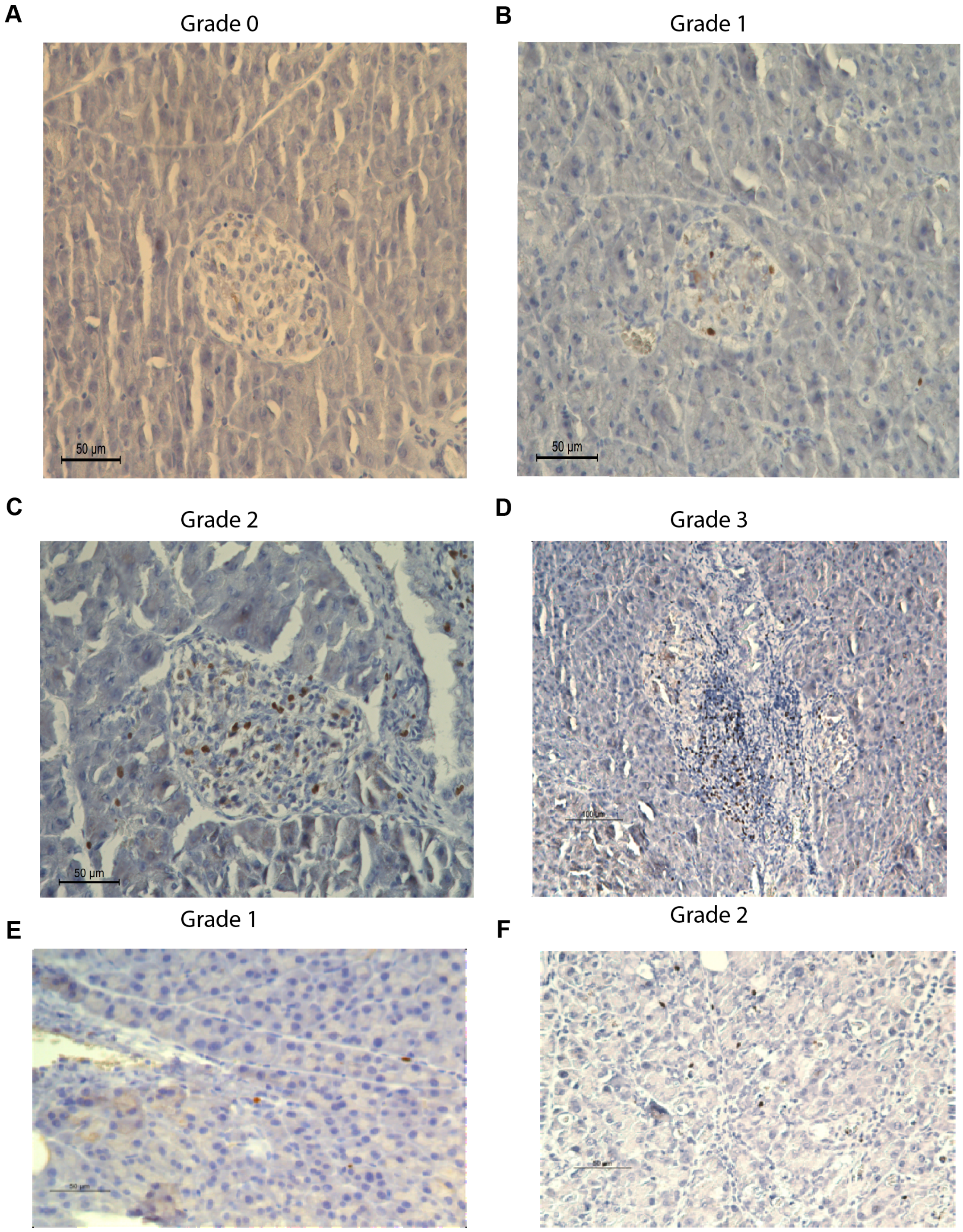

Supplementary Figure 3

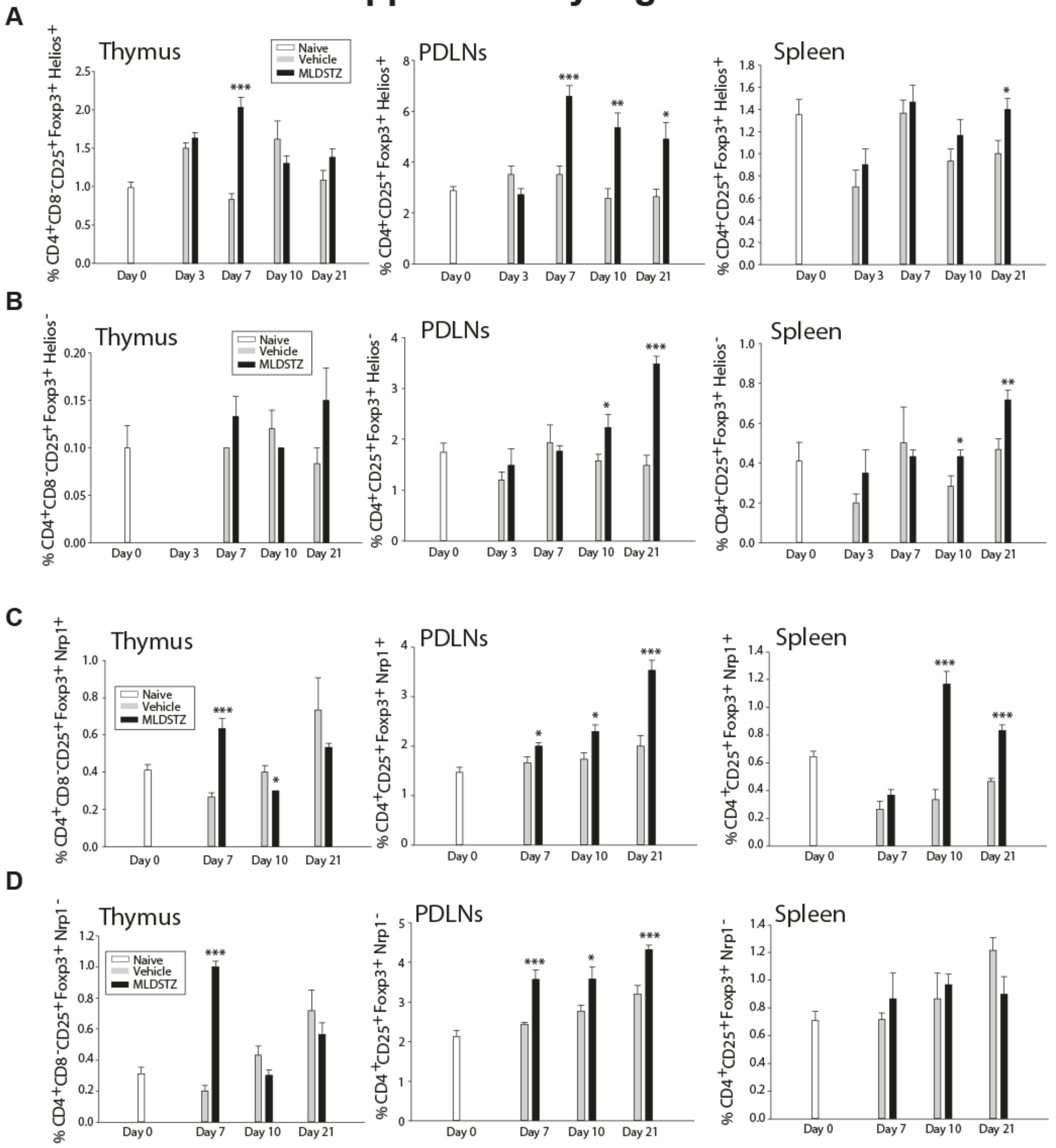

Supplementary Figure 4

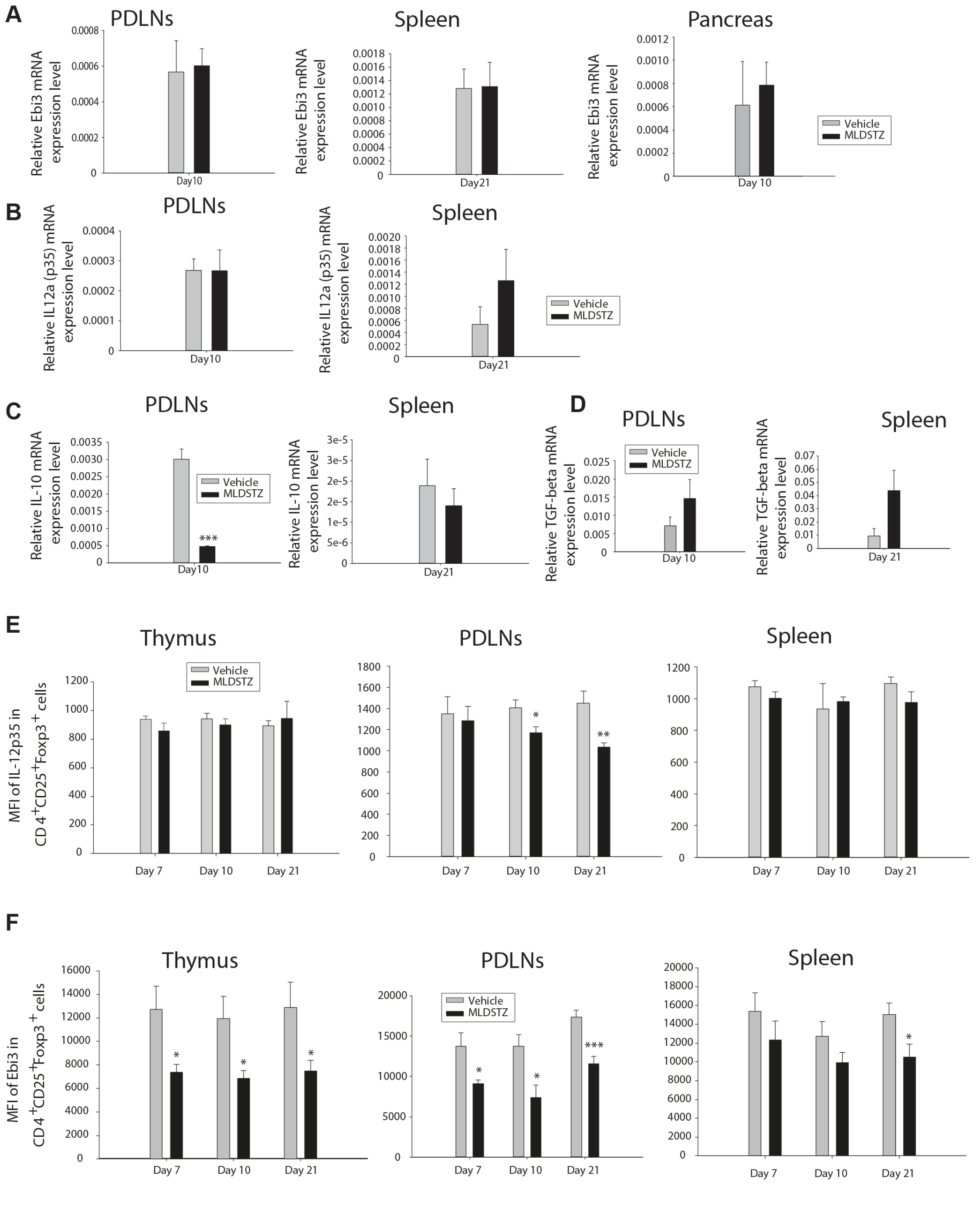

Supplementary Figure 5

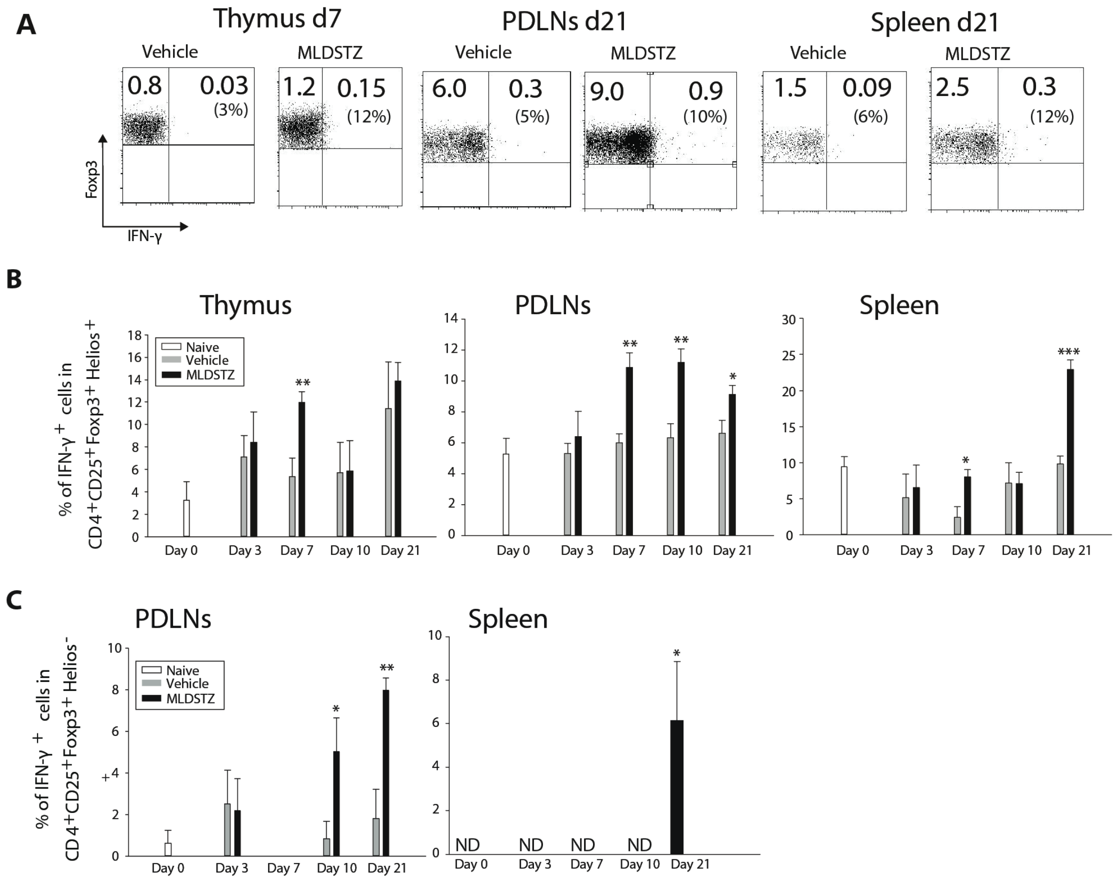

Supplementary Figure 6

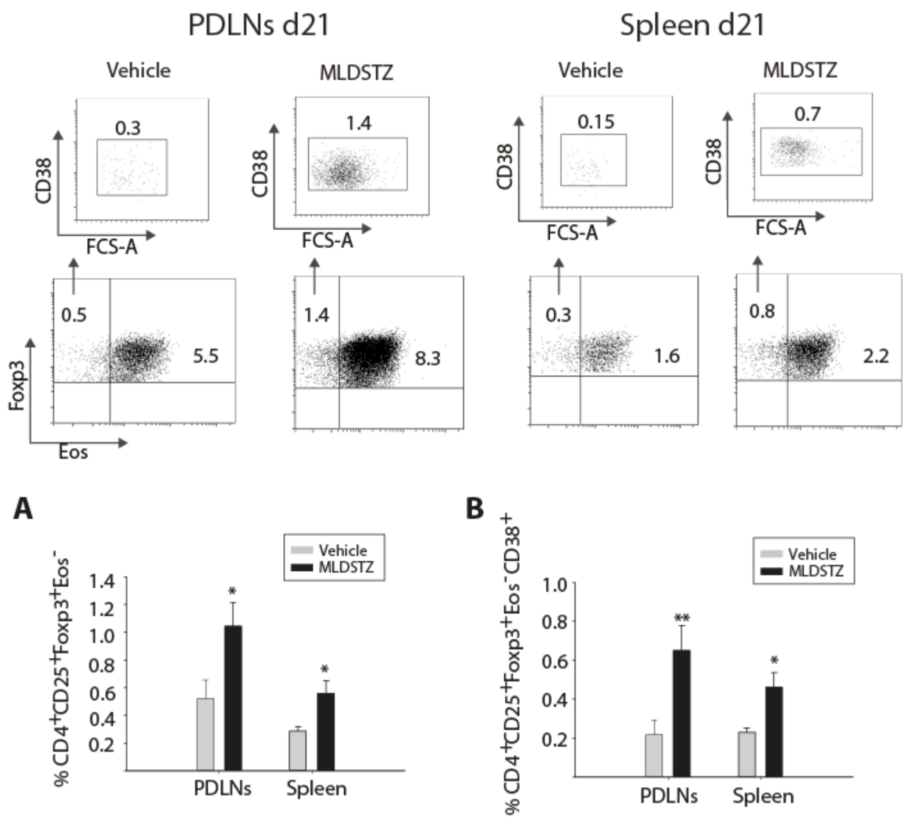

Supplementary Figure 7

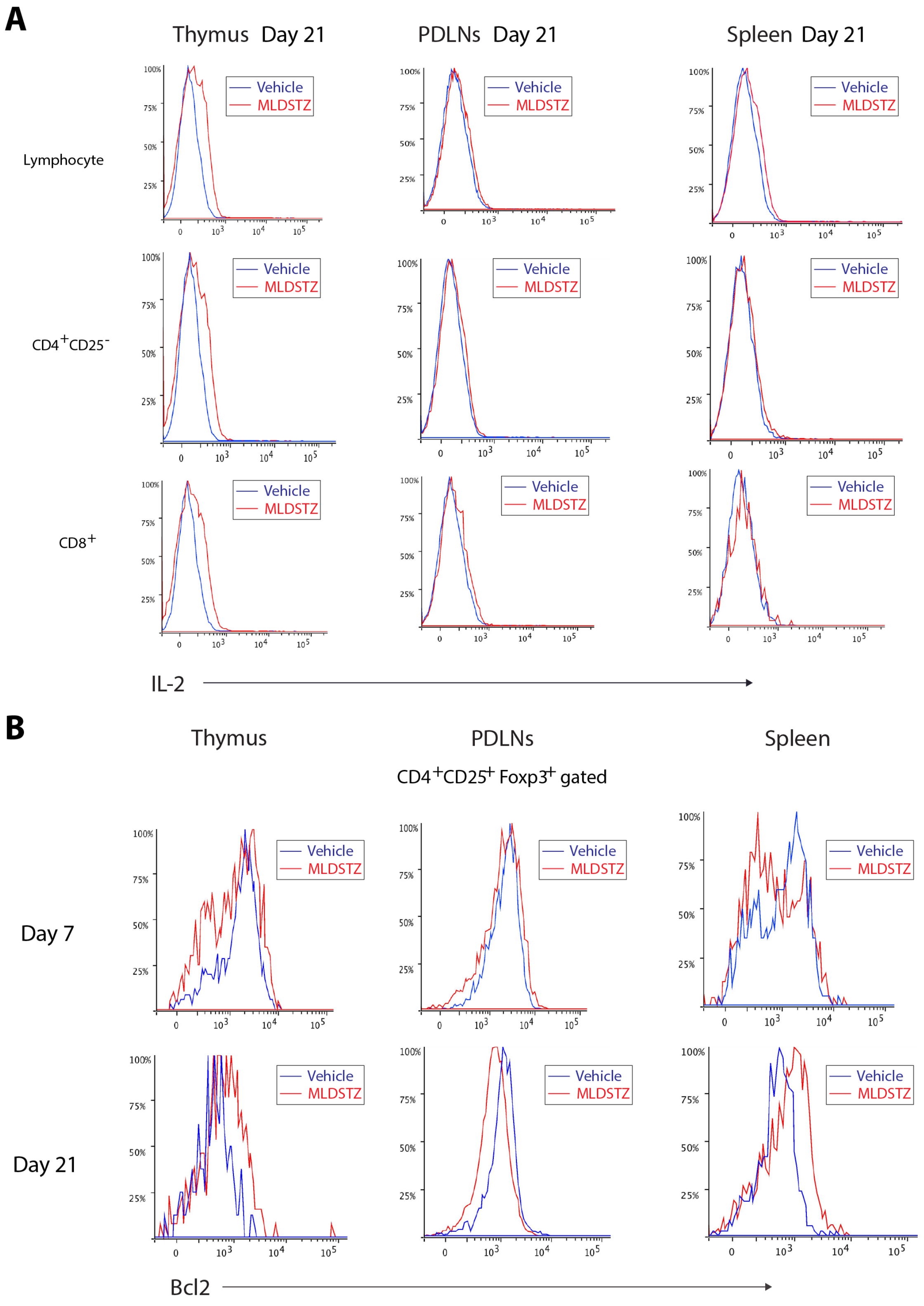

Supplementary Figure 8

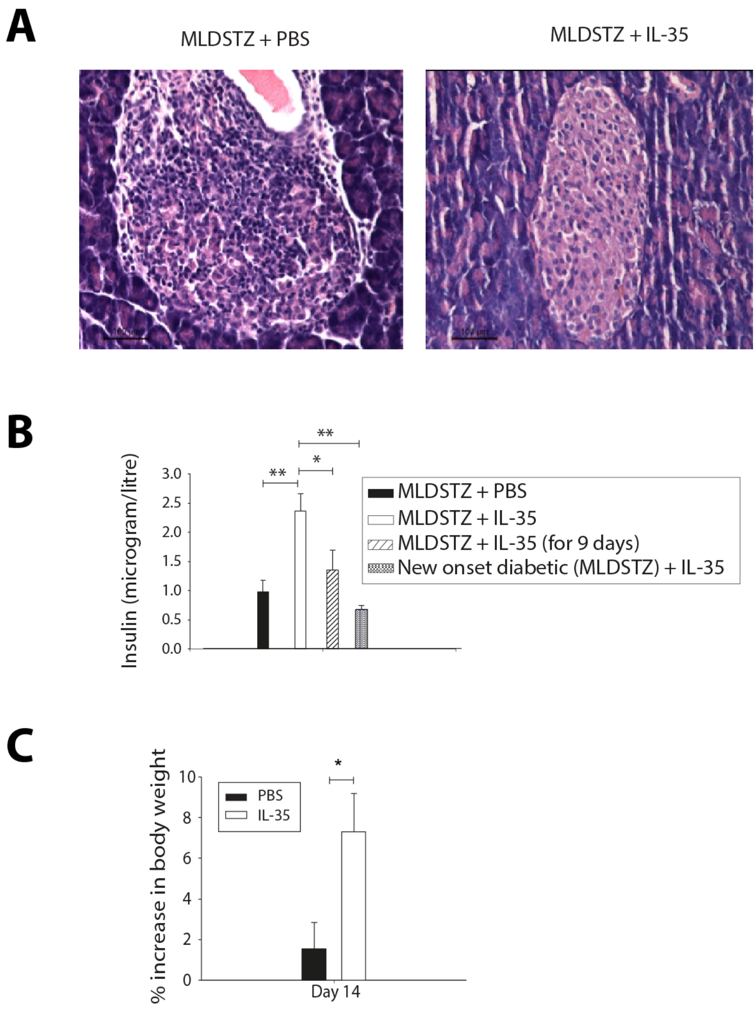

Supplementary Figure 9

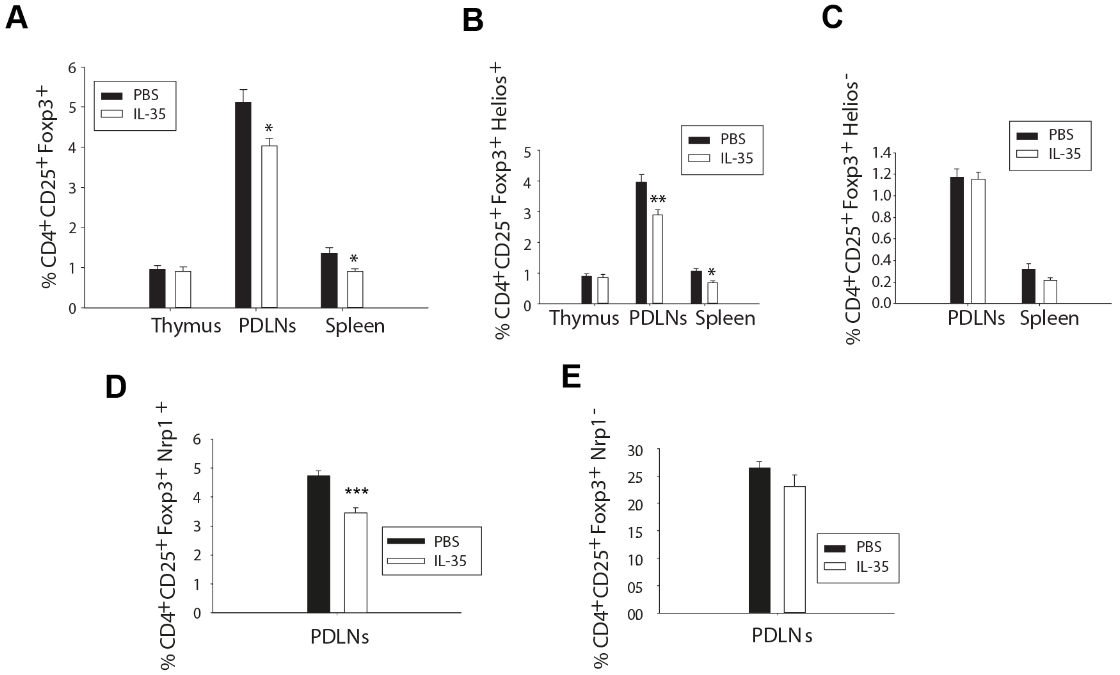

Supplementary Figure 10

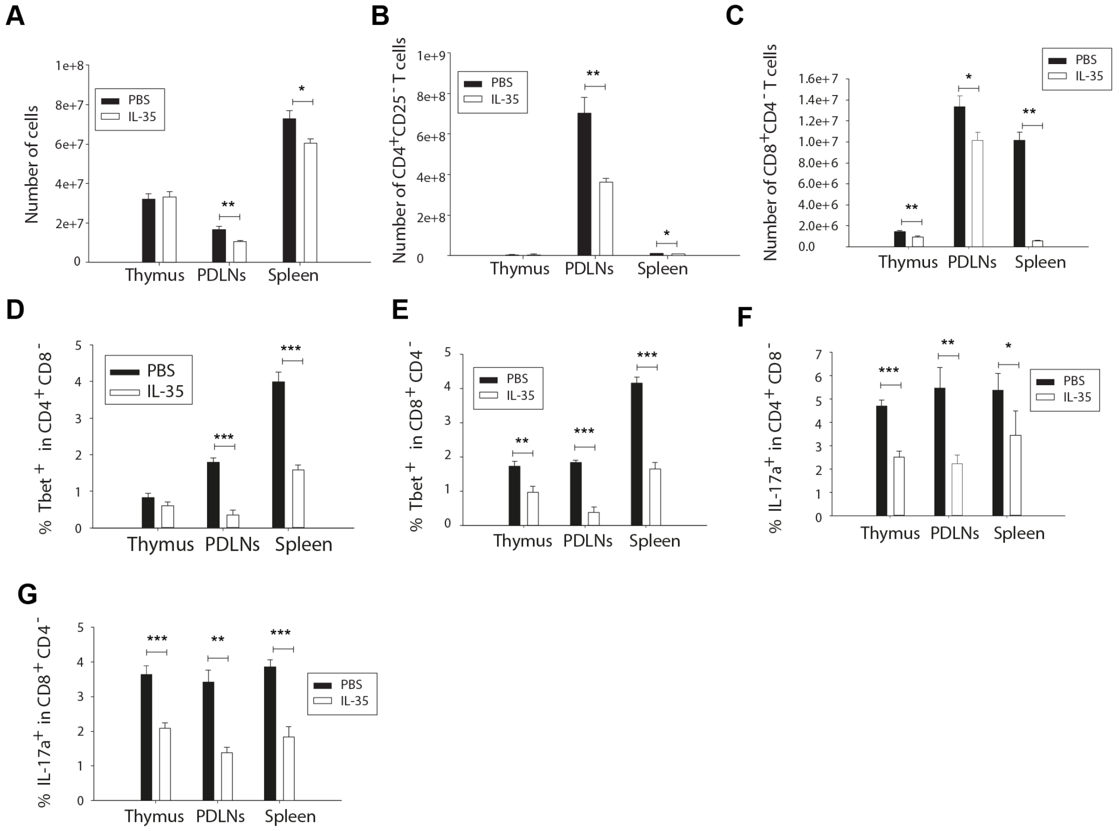

Supplementary Figure 11

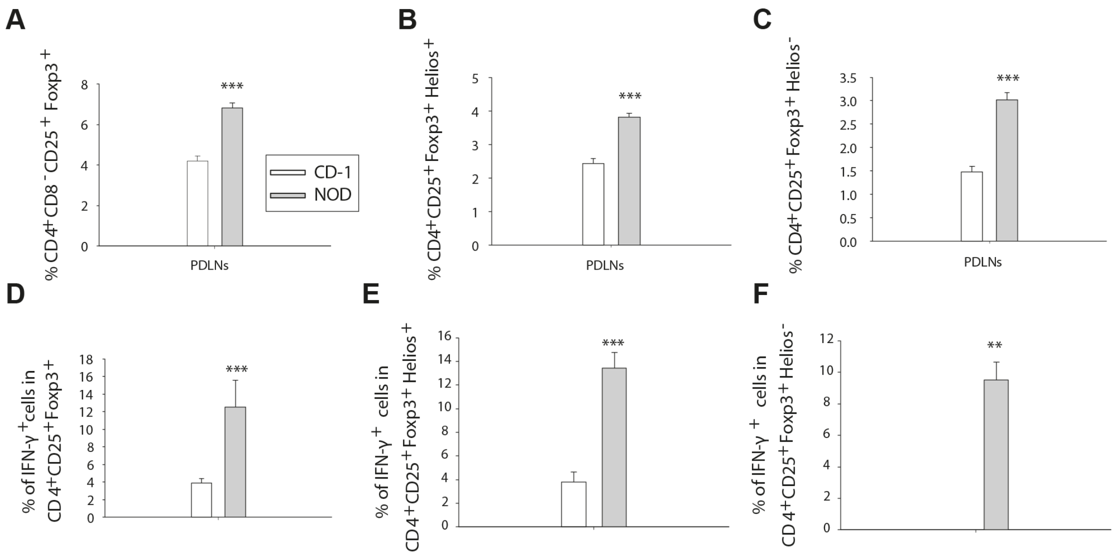

Supplementary Figure 12

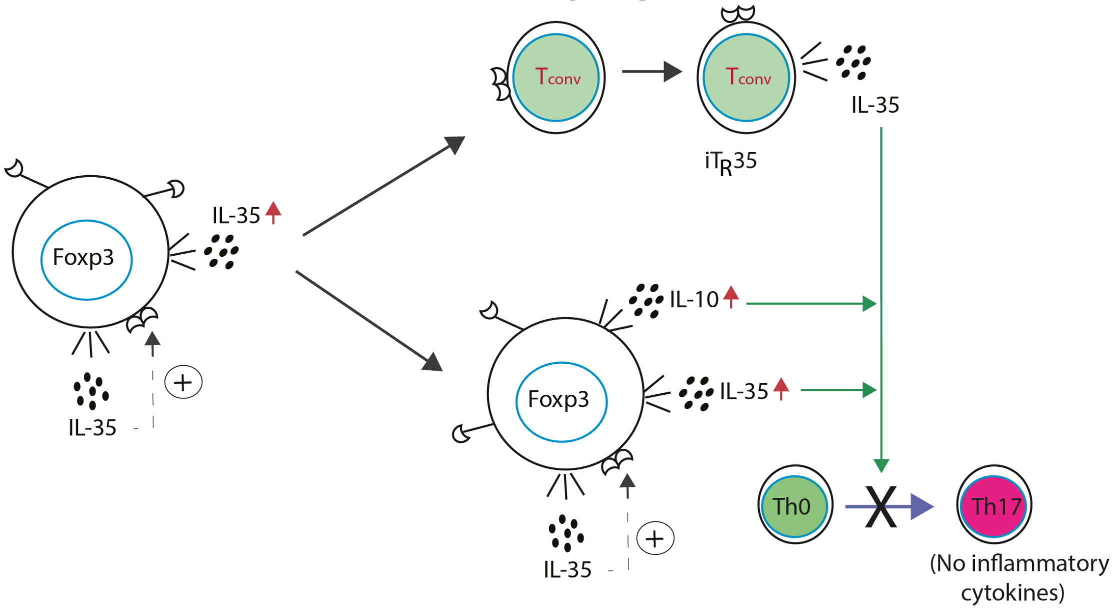

Supplementary table 1. Morphological analysis of pancreas and semi-quantitative analysis of Foxp3$^+$ cells in pancreas.

Haematoxylin and eosin stained sections of pancreata were analyzed for insulitis by scoring (0, 1, 2, 3 and 4) and the numbers of Foxp3$^+$ by scoring (0, 1,2 and 3) as described in the Methods section. The mode values are expressed ($n = 6$). Mann-Whitney Rank Sum tests were performed for comparisons between vehicle and MLDSTZ treated groups on corresponding days. *, ** and *** denote $p < 0.05$, $p < 0.01$, and $p < 0.001$, respectively.

On day 14 the insulitis scoring was performed on Foxp3 stained section instead of hematoxylin and eosin stained tissue sections.

Mouse number/Days	Insulitis		Foxp3^{+} cells in endocrine tissue		Foxp3^{+} cells in exocrine tissue	
	Vehicle	MLDSTZ	Vehicle	MLDSTZ	Vehicle	MLDSTZ
Day 0						
1	0		0		0	
2	0		0		0	
3	0		0		0	
4	0		0		0	
5	0		0		0	
6	0		0		0	
7	0		0		0	
Day 3						
1	0	0	0	0	1	0
2	0	0	0	0	0	0
3	0	0	0	0	0	0
4	0	0	1	0	0	1
5	0	0	0	0	2	1
6	0	0	0	1	0	0
Day 7				***		
1	0	1	0	1	0	0
2	0	0	0	0	0	0
3	0	0	0	0	0	0
4	0	0	0	0	0	1
5	0	1	0	1	0	1
6	0	1	0	2	0	1
Day 10		*				**
1	0	1	0	0	0	1
2	0	2	1	2	0	1
3	0	0	0	0	0	1
4	0	1	0	2	0	1
5	0	1	0	2	0	1
6	0	1	0	1	0	1
Day 14[#]		**				**
1	0	2	0	1	0	1
2	0	2	0	0	0	1
3	0	1	0	0	0	0
4	0	2	0	3	0	1
5	0	1	0	2	0	1
6	0	2	0	1	0	1
Day 21		**				**
1	0	2	0	1	0	1
2	0	2	0	1	0	1
3	0	2	0	0	0	1
4	0	2	0	0	0	0
5	0	2	0	0	0	1
6	0	2	0	2	0	1

Supplementary table 2. The numbers of Foxp3$^+$ cells are increased in pancreata of pre-diabetic NOD female mice

Haematoxylin and eosin stained sections of pancreata were analyzed for insulitis by scoring (0, 1, 2, 3 and 4) and the numbers of Foxp3$^+$ by scoring (0, 1,2 and 3) as described in the Methods section. The mode values are expressed (n = 6). Mann-Whitney Rank Sum tests were performed for comparisons between vehicle and MLDSTZ treated groups on corresponding days.

Insulitis		Foxp3$^+$ cells in endocrine tissue		Foxp3$^+$ cells in exocrine tissue	
CD-1	NOD	CD-1	NOD	CD-1	NOD
0	3	0	3	0	1
0	3	0	3	0	1
0	3	0	3	0	1
0	3	0	3	0	1
0	3	0	3	0	0
0	3	0	3	0	1
P = 0.002		P = 0.002		P = 0.015	

Supplementary table 3. The degree of insulitis is decreased and insulin staining score is increased in pancreata of IL-35 treated NOD female mice.

Insulin stained sections of pancreata of PBS (200 μl, i.p.) or IL-35 (0.75 μg daily, i.p.) treated mice were analyzed for insulitis by scoring (0, 1, 2, 3 and 4) and insulin by scoring (0, 1, 2 and 3) as described in the Methods section.

Insulitis		Insulin staining	
PBS	IL-35	PBS	IL-35
4	3	0	0
4	3	0	0
4	2	0	1
4	0	0	3
4	0	0	3
4	0	0	3
P = 0.002		P = 0.06	

Supplementary table 4

The sequences of the primers used in RT-PCR analysis for quantification of different genes expression in PDLNs and spleen cells, and in pancreas.

Gene name	Primer	Sequences	GeneBank number
Foxp3	Forward	TTATCCGATGGGCCATCCT	#NM_054039
	Reverse	CAAAGCACTTGTGCAGGCTC	
IL-12p35	Forward	CAATCACGCTACCTCCTCTTT	#NM_001159424
	Reverse	AGTTTTTCTCTGGCCGTC	
Ebi3	Forward	CCTCTCCAGGCTCCCAACT	#NM_015766
	Reverse	GAGGGTCCGGCTTGATGATT	
IL-17	Forward	CTGGACTCTCCACCGCAA	#NM_010552
	Reverse	CAGCTTTCCCTCCGCAT	
IL-10	Forward	CATTCATGGCCTTGTAGACACC	#NM_010548
	Reverse	CTTAATGCAGGACTTTAAGGGTTA	
TGF-β	Forward	GGACACACAGTACAGCAAGGTC	#AJ_0009862
	Reverse	TCAGCTGCACTTGCAGGAG	
β-actin	Forward	CCGTGAAAAGATGACCCAGAT	#EF_095208.1
	Reverse	CTCAGCTGTGGTGGTGAAGC	

Supplementary table 5. Descriptive data of healthy controls (n=13) and patients with recent onset type 1 diabetes (T1D) (n=8) and long-standing T1D (n=19). Values are presented as mean ± SEM. ** denotes $p<0.01$ and *** $p<0.001$ when compared to patients with recent onset T1D. ### denotes $p<0.001$ when compared to healthy controls.

**Variable**	**Healthy controls (n=13)**	**Recent-onset T1D (n=8)**	**Long-standing T1D (n=19)**
**Female (n, %)**	9 (69%)	4 (50%)	13 (68%)
**Age (years)**	26.4 ± 1.1	22.8 ± 3	29.6 ± 1.9
**Age at onset (years)**	n/a	21.9 ± 2.9	11.4 ± 1.9 **
**Disease duration (months)**	n/a	9.1 ± 2	225 ± 23 ***
**Fasting blood glucose (mmol/l)**	5.04 ± 0.16	8.1 ± 1.5	11.7 ± 1.3 ###
**BMI (kg/m^2)**	22.5 ± 0.5	22.8 ± 1.2	24.8 ± 0.7
**GAD positive (%)**	n/d	8 (100%)	14 (74%)
**IA2 positive (%)**	n/d	7 (88%)	11 (58%)
